# Nighttime light dynamics reveal peri-urban brightening and population decoupling in the Chengdu Chongqing megaregion

**DOI:** 10.1038/s41598-025-34706-9

**Published:** 2026-01-10

**Authors:** Wenli Liang, Rui Liu, Pinglang Kou

**Affiliations:** 1 State Key Laboratory of Geohazard Prevention and Geoenvironment Protection, Chengdu University of Technology, Chengdu, 610059 Sichuan China; 2 Chongqing Key Laboratory of GIS Application, School of Geography and Tourism, Chongqing Normal University, Chongqing, 400047 China; 3 Chongqing Engineering Research Center of Spatial Big Data Intelligent Technology, Chongqing University of Posts and Telecommunications, Chongqing, 400065 China; 4 Key Laboratory of Tourism Multisource Data Perception and Decision, Ministry of Culture and Tourism (TMDPD, MCT), Chongqing University of Posts and Telecommunications, Chongqing, 400065 China; 5 No. 12, Tianchen Road, Shapingba District, Chongqing, 400047 P.R. China

**Keywords:** Night-time light, Urban expansion, Peri-urban ring, Chengdu–Chongqing economic circle, Remote sensing, Environmental studies, Geography, Geography

## Abstract

Night-time light (NTL) composites offer a unique spatiotemporal view of urban growth by capturing electricity consumption, infrastructure density, and economic activity patterns that traditional land-cover surveys cannot fully reveal, yet inland China’s megaregions remain understudied from this perspective. Using a 23-year (2000–2022) cross-calibrated NTL product integrated with land-cover, population, and topographic data, we analysed illumination dynamics in the Chengdu–Chongqing economic circle. Mean radiance rose by 78% while the lit area expanded from 0.53% to almost 12%. About three-quarters of this growth is confined to a 4–6 km peri-urban “neon ring” created chiefly by cropland conversion to high-rise housing and logistics estates, now ~ 40% brighter than historic cores. Targeted LED retrofits and façade-lighting curfews produced persistent downtown “dark hollows”, proving that local policy can dampen sky-glow. Cropland-to-urban parcels emitted roughly 6 nW cm⁻² sr⁻¹ more light than other transitions, and protected wetlands brightened threefold, flagging covert development. Spatial autocorrelation analysis revealed extremely high global Moran’s I values (> 0.92 after 2013), indicating strong spatial clustering patterns of illumination changes, with local LISA analysis identifying hotspots predominantly concentrated in the core areas of both cities. Projections indicate ~ 1,000 km² of additional luminous sprawl by 2042 as radiance continues to climb despite a 1.6% population decline, underscoring a widening decoupling between light and headcount.

**Supplementary Information:**

The online version contains supplementary material available at 10.1038/s41598-025-34706-9.

## Introduction

Urban areas are expected to accommodate almost seventy per cent of the world’s population by 2050, yet the pace, pattern, and environmental footprint of this expansion remain unevenly documented, particularly across the interior regions of China^1,2^. Satellite-derived nighttime light (NTL) composites offer a unique perspective on urban growth, as radiance reflects electricity consumption, infrastructure density, and economic activity^3–6^. Despite extensive research on China’s eastern megacities, studies on the Chengdu-Chongqing economic circle remain sparse^7,8^. This corridor spans the low-relief Chengdu Plain and the deeply incised headwaters of the Yangtze River, adding more than fifteen million residents after 2000, and doubling its gross regional product, making it an ideal natural laboratory for investigating how topography, land-cover turnover, and demographic redistribution jointly reshape the nocturnal skyline^9,10^.

Two persistent challenges have hindered a clear understanding of these dynamics. First, the low-resolution DMSP-OLS sensor saturates at radiance levels typical of Chinese city centres, while the higher-dynamic-range VIIRS Day/Night Band introduces a post-2012 discontinuity, complicating trend analyses^11,12^. Second, most cross-sensor products do not integrate ancillary datasets such as land-cover mosaics, high-resolution topography, and gridded population counts, which are essential for separating socioeconomic drivers from geomorphic constraints^13–15^. Moreover, existing studies lack systematic spatial statistical methods to quantify spatial clustering patterns and heterogeneity characteristics of urban expansion, making it difficult to identify statistically significant hotspots and coldspots, thus limiting in-depth understanding of spatial processes of urbanisation at the regional scale^14^. As a result, existing narratives alternately portray Chengdu and Chongqing as a single coalescing megaregion or as two competitively brightening poles, without resolving the finer-scale gradients that would test these competing hypotheses^16^. Furthermore, no prior study has systematically examined phenomena such as peri-urban brightening rings, policy-induced downtown dimming, or population-light decoupling in this region using an integrated multi-source framework combining NTL, land-cover, population, and topographic data with spatial autocorrelation methods.

To address these shortcomings, we assemble a twenty-three-year cross-calibrated NTL archive for 2000–2022 that harmonises DMSP-OLS and VIIRS radiances at five-hundred-metre resolution, following the sensor-coupling protocol of the Yangtze River Delta Science Data Centre^1,17^ and the extended NPP-VIIRS time series methodology^18^. We fuse this backbone with MODIS land-cover transitions, LandScan one-kilometre population grids, and thirty-metre Shuttle Radar Topography Mission elevations. Using these inputs, we first quantify the spatial and temporal heterogeneity of NTL brightening and dimming across the corridor. Second, we isolate the relative influence of cropland conversion, wetland encroachment, and slope breaks on radiance trajectories. Third, we project urban-boundary migration and per-capita luminosity through 2042 with gradient-aware time-series models^7,16^. The resulting multivariate, topographically explicit chronology offers the most granular and policy-relevant account to date of how infrastructure policy, geomorphic setting, and demographic flux converge to redefine the night sky over one of Asia’s fastest-growing megaregions.

## Methods

### Study area

The Chengdu–Chongqing Dual-City Economic Circle (CCEC) is the largest inland urban agglomeration in Southwest China, spanning approximately 185,000 km² and straddling two distinct geomorphic regions: the low-relief Chengdu Plain and the more rugged Sichuan-Chongqing fold belt. This extent is comparable to or exceeds other well-studied urban agglomerations such as the Yangtze River Delta (~ 110,000 km²) and the Pearl River Delta (~ 55,000 km²). At 1-km NTL resolution, the study area yields approximately 185,000 pixels annually and over 4.25 million spatiotemporal observations across the 23-year record, providing sufficient data for robust statistical analysis while maintaining a scale appropriate for detecting fine-grained spatial phenomena. This study focuses on the CCEC, which has experienced rapid urban expansion and economic growth, particularly in the cities of Chengdu and Chongqing. The region’s climate is characterised by a humid subtropical monsoon, with annual temperatures averaging 16–18 °C and rainfall around 1,000 mm, supporting intensive agriculture on the plains and terraced farming in the surrounding mountainous areas^1^.

**Fig. 1:**
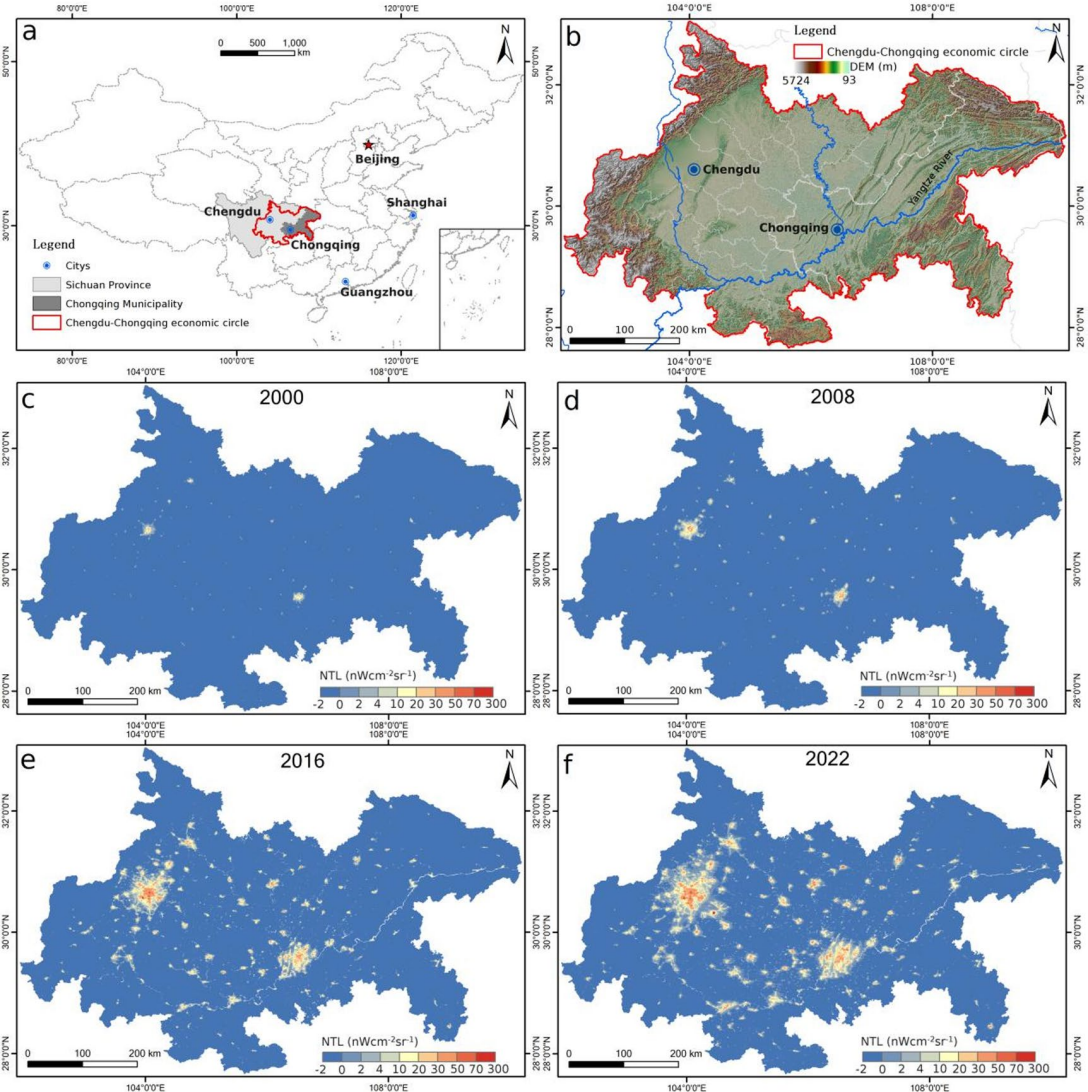
Location and NTL changes of the study area. (**a**) The study area in China; (**b**) The digital elevation model (DEM) of the study area. (**c-f**) The NTL of the study area in 2000, 2008, 2016, and 2022, respectively.

As shown in Fig. 1, the study area includes both the flat Chengdu Plain and the more mountainous terrain of the Sichuan-Chongqing fold belt, with Chengdu and Chongqing serving as the central urban hubs of the region (Fig. 1b). The CCEC faces a growing challenge of excessive urban development, particularly in areas where natural landscapes, such as protected wetlands, are being encroached upon. These wetlands, nominally off-limits to development, have seen significant increases in nighttime light (NTL) intensity since 2005, suggesting covert developments, such as dredging and land reclamation, that evade traditional land-cover assessments^14^. Figure 1c-f illustrates the evolution of NTL in the region over time, highlighting the rapid expansion of urban areas from 2000 to 2022.

### Data acquisition and preprocessing

**Fig. 2:**
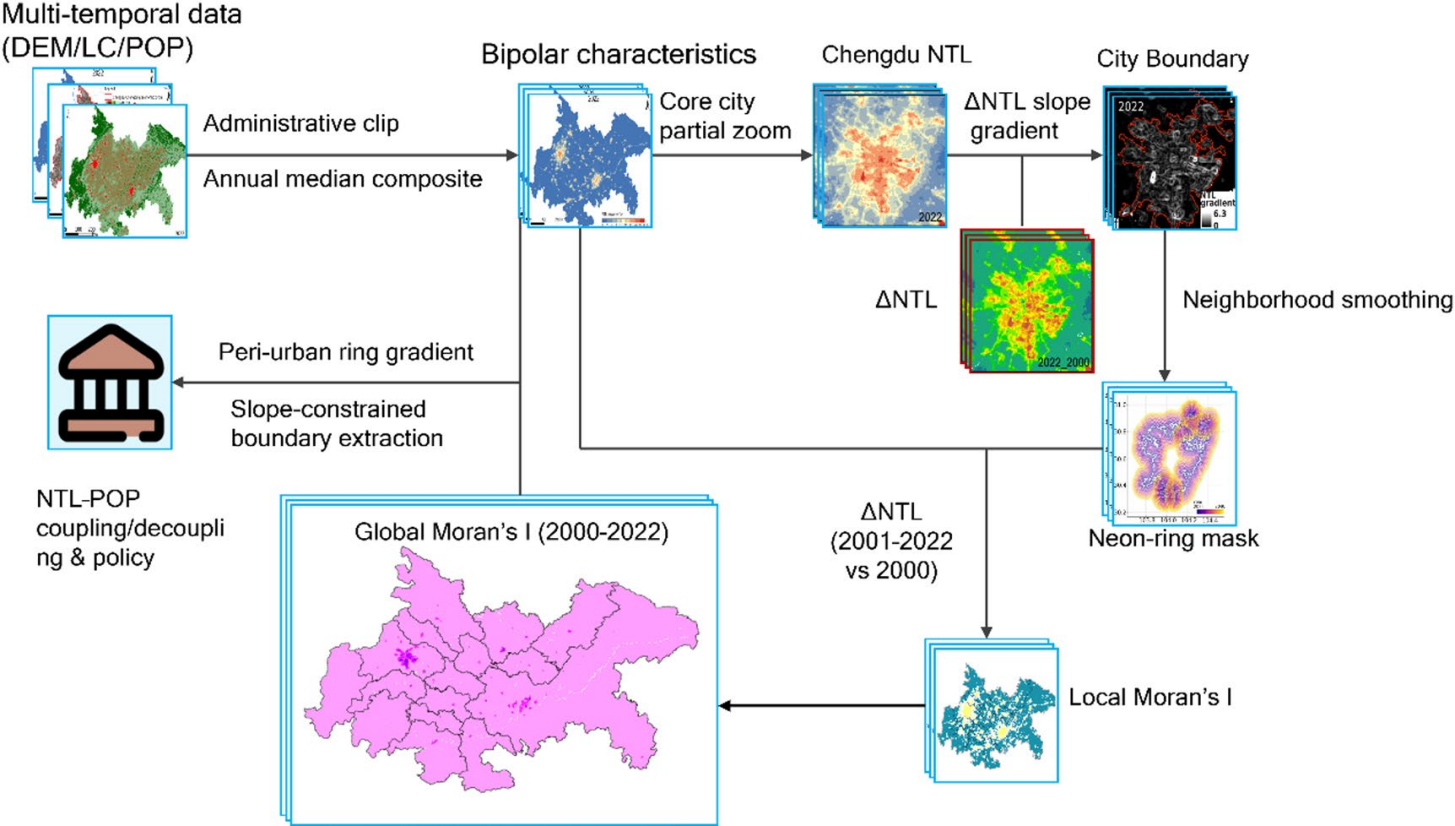
Flowchart of the Research Methodology.

This study integrated multi-source remote-sensing and ancillary geographic datasets spanning 2000–2022 (Table 1). All datasets underwent systematic preprocessing and quality control to ensure spatial consistency and temporal comparability. We also visualised the data-processing and analytical methodology with a flowchart (Fig. 2).

**Table 1:** Summary of datasets used in this Study.

Data Type	Source	Temporal Coverage	Spatial Resolution	Format	Access	Primary Use
Nighttime Light Data	Yangtze River Delta Science Data Centre, DMSP-OLS-like Product	2000–2022	1 km	GeoTIFF	http://geodata.nnu.edu.cn/; DOI: 10.12041/geodata.54416522887785.ver2.db	NTL intensity analysis, spatiotemporal dynamics, trend projection
Land Cover Data	MODIS Land Cover Type (MCD12Q1), NASA LAADS DAAC	2001, 2022	500 m	HDF	https://ladsweb.modaps.eosdis.nasa.gov/	Land-use transition analysis; NTL increment by land-cover type
Population Data	LandScan Global Population Database, Oak Ridge National Laboratory	2000–2022	1 km (≈ 30 arc-seconds)	GeoTIFF	https://landscan.ornl.gov/	Population-density mapping, per-capita NTL calculation, population–light relationship
Topographic Data	SRTM Digital Elevation Model (DEM), USGS Earth Explorer	2000	30 m	GeoTIFF	https://earthexplorer.usgs.gov/	Terrain-gradient calculation, urban-boundary extraction, slope analysis
Administrative Boundaries	National Geomatics Centre of China	2020	Vector	Shapefile	https://www.ngcc.cn (official website)	Study-area delineation, spatial overlay analysis

1. Night-time light (NTL) data. We used the 1-km “DMSP-OLS-like” China NTL dataset (1992–2024) from the Yangtze River Delta Science Data Centre (DOI: 10.12041/geodata.54416522887785.ver2.db). The dataset had calibrated DMSP-OLS observations (1992–2013) using the pseudo-invariant features (PIF) method, in which temporally stable pixels (e.g., deserts, perennial glaciers) served as references to build calibration equations and remove sensor degradation, gain fluctuation, and system biases. Before annual SNPP-VIIRS compositing, the dataset applied spatiotemporal interpolation to repair missing values in monthly inputs (2013–2024), ensuring temporal continuity and spatial completeness. Data were provided as GeoTIFF at 1-km resolution and had been widely validated in urban expansion and socioeconomic studies. We downloaded annual layers for 2000–2022 for spatiotemporal analyses. We directly extracted the radiance values (in nW cm⁻² sr⁻¹) for each pixel from the calibrated NTL dataset and calculated statistical metrics (mean, sum, etc.) across all pixels in the study area to quantify spatiotemporal changes in NTL intensity.
2. Land cover data. Land cover information was obtained from the MODIS Land Cover Type product (MCD12Q1; 500 m) via NASA LAADS DAAC for 2001 and 2022. We adopted the IGBP scheme and reclassified the 17 native classes into six categories: forest, grassland, wetland, cropland, urban, and bare land. Reclassification was performed in ArcGIS 10.7 and resampled to 1 km using nearest-neighbour to match the NTL grid.
3. Population data. We used the LandScan global population database (Oak Ridge National Laboratory), which provided annual population distribution grids at ~ 1-km resolution (2000–2022) derived from the ambient (daytime) population model integrating census data, land use, road networks, night-time lights, and other geospatial inputs. Annual rasters for the study area were obtained from the official portal and reprojected to WGS84 for spatial statistics.
4. Topographic data. We used Shuttle Radar Topography Mission (SRTM) 30-m digital elevation model (DEM) tiles downloaded from USGS Earth Explorer. The DEM supported slope derivation, identification of topographic lineaments, and assisted urban boundary extraction. Slope and terrain analyses were conducted with ArcGIS Spatial Analyst.
5. Administrative boundaries. Vector administrative boundaries were obtained from the National Geomatics Center of China to delineate the Chengdu–Chongqing economic circle and support overlay analyses.
6. Common preprocessing. Before analysis, all datasets (i) were unified to the WGS84 geographic coordinate system; (ii) were resampled to a common 1-km resolution using nearest-neighbour; (iii) underwent co-registration checks (positional error < 0.5 pixel); and (iv) were screened and corrected for nodata and outliers. Data processing was performed in Python 3.8 (GDAL, Rasterio, NumPy) and ArcGIS 10.7.

### NTL dynamics and Spatial analysis

We calculated annual mean and standard deviation of NTL intensity using Python and constructed error bars representing 0.5 times the standard deviation. A linear regression model analysed temporal trends and projected long-term patterns from 2023 to 2042. The proportion of lit area was calculated by dividing the number of NTL pixels greater than zero in each year by the total number across 2000–2022:

1 $$\:\begin{array}{c}{P}_{t}=\frac{{N}_{t}}{\sum\:_{t=2000}^{2022}{N}_{t}}\end{array}$$

where $\:{\mathrm{P}}_{\mathrm{t}}$ represents the proportion in year $\:\mathrm{t}$, and $\:{\mathrm{N}}_{\mathrm{t}}$ denotes pixels with NTL values greater than zero.

Annual NTL increment was calculated as:

2 $$\:\begin{array}{c}{\Delta\:}NTL(x,y,t)=NTL(x,y,t)-NTL(x,y,2000)\end{array}$$

We applied linear regression to quantify temporal trends:

3 $$\:NT{L}_{t}=\alpha\:+\beta\:t+\epsilon\:$$

achieving $\:{\mathrm{R}}^{2}>0.89$ for the study region. Frequency distribution analysis identified occurrence probabilities of different intensity increments across years.

To quantitatively assess spatial clustering patterns, we conducted comprehensive spatial autocorrelation analyses using PySAL. Global Morans I measured overall spatial autocorrelation:

4 $$\:\begin{array}{c}I=\frac{n\sum\:_{i}\sum\:_{j}{w}_{ij}({x}_{i}-\overline{x})({x}_{j}-\overline{x})}{\sum\:_{i}\sum\:_{j}{w}_{ij}\sum\:_{i}({x}_{i}-\overline{x}{)}^{2}}\end{array}$$

where $\:\mathrm{n}$ is the number of spatial units, $\:{\mathrm{x}}_{\mathrm{i}}$ and $\:{\mathrm{x}}_{\mathrm{j}}$ are NTL increment values, $\:\overline{\mathrm{x}}$ is the mean, and $\:{\mathrm{w}}_{\mathrm{i}\mathrm{j}}$ are spatial weights. We constructed a Queen contiguity spatial weights matrix and implemented 30% random sampling for computational efficiency ($\:\mathrm{G}\mathrm{L}\mathrm{O}\mathrm{B}\mathrm{A}\mathrm{L}\_\mathrm{S}\mathrm{A}\mathrm{M}\mathrm{P}\mathrm{L}\mathrm{E}\_\mathrm{P}=0.3$), with statistical significance evaluated through 199 Monte Carlo permutations.

Local Indicators of Spatial Association (LISA) identified specific clustering locations through Local Moran’s I:

5 $$\:\begin{array}{c}{I}_{i}=\frac{({x}_{i}-\overline{x})}{{m}_{2}}\sum\:_{j}{w}_{ij}({x}_{j}-\overline{x})\end{array}$$

Where

6 $$\:{m}_{2}=\frac{\sum\:_{i}({x}_{i}-\overline{x}{)}^{2}}{n}$$

We classified pixels into five categories: high-high clusters (hotspots), low-low clusters (coldspots), high-low outliers, low-high outliers, and not significant. Given computational demands, we employed a downsampling-upsampling strategy ($\:\mathrm{L}\mathrm{I}\mathrm{S}\mathrm{A}\_\mathrm{D}\mathrm{O}\mathrm{W}\mathrm{N}\mathrm{S}\mathrm{A}\mathrm{M}\mathrm{P}\mathrm{L}\mathrm{E}=4$), reducing computational time approximately 16-fold. All analyses used fixed random seeds ($\:\mathrm{S}\mathrm{E}\mathrm{E}\mathrm{D}=0$) for reproducibility.

### NTL-Population-Land cover integration

To examine relationships between NTL increments and baseline illumination, we categorised changes into positive (brightening) and negative (dimming) by calculating differences between 2022 and 2001 values. We computed area proportions, mean values, median values, and standard deviations, and performed correlation analyses between baseline NTL (2001) and subsequent increments using SPSS 20.0.

NTL is an indicator of human activity and economic development^3–5,15^, as radiance reflects electricity consumption, infrastructure density, and economic activity levels, and also serves as an effective method to assess land use change^13,14^. While some studies set NTL values to zero for non-urban land covers to mitigate blooming effects, our analysis focuses on land-cover transitions rather than static categories, tracking NTL changes for the same pixels before and after conversion to capture genuine urbanization signals.

We integrated MODIS land cover data (2001, 2022) reclassified into six types: forest, grassland, wetland, cropland, urban, and barren. Land cover transition matrices analyzed changes and proportions, with NTL increments grouped by land cover type. We calculated mean and standard deviation for both all values and non-zero values to exclude area-difference effects, using R software for analysis.

To compare Chengdu and Chongqing, we analysed positive NTL increment frequency distributions at different brightness levels and evaluated overall variations using box plots. Linear regression models projected 20-year trends. We employed two-way ANOVA with city and land cover type as factors, testing whether mean NTL increments differed across groups:

7 $$\:\begin{array}{c}F=\frac{M{S}_{between}}{M{S}_{within}}\end{array}$$

Spatial analysis was performed in ArcGIS 10.7 (Extract by Mask, Zonal Statistics, raster overlay), with statistical testing in SPSS 20.0.

### Urban boundary evolution and population coupling

We analyzed urban spatial structure using NTL data from 2000 to 2022. By extracting brightest pixels and performing slope analysis, we identified urban centres, edges, and boundaries. The Contour List tool in ArcGIS 10.7 delineated urban-rural boundaries with city-specific thresholds, followed by boundary smoothing and topology checks. Annual NTL gradients were calculated using spatial interpolation and edge detection, with frequency distributions quantifying characteristics. Time series analysis examined gradient trends and linear regression projected 20-year futures.

Urban boundary evolution was forecasted using a spatiotemporal model simulating expansion by fixed annual ratios (0.5%). While this assumes uniform expansion without accounting for planning or environmental factors, it provides insights into historical evolution and future development references.

We investigated population–NTL relationships using LandScan data (see Table 1). While 1-km resolution may introduce uncertainties at finer scales, it is appropriate for regional and city-scale analyses. Using ArcGIS, we: (1) calculated correlations between annual population and NTL changes; (2) performed trend analysis and predicted future patterns; (3) compared Chengdu–Chongqing relationships to explain urban light diffusion patterns. We computed the annual mean and standard deviation of population and per capita NTL intensity (2000–2022), applied linear regression to forecast 20-year trends, and introduced error bands of 0.1 times the standard deviation to quantify uncertainty.

## Results

### Temporal and Spatiotemporal dynamics of NTL intensity

**Fig. 3:**
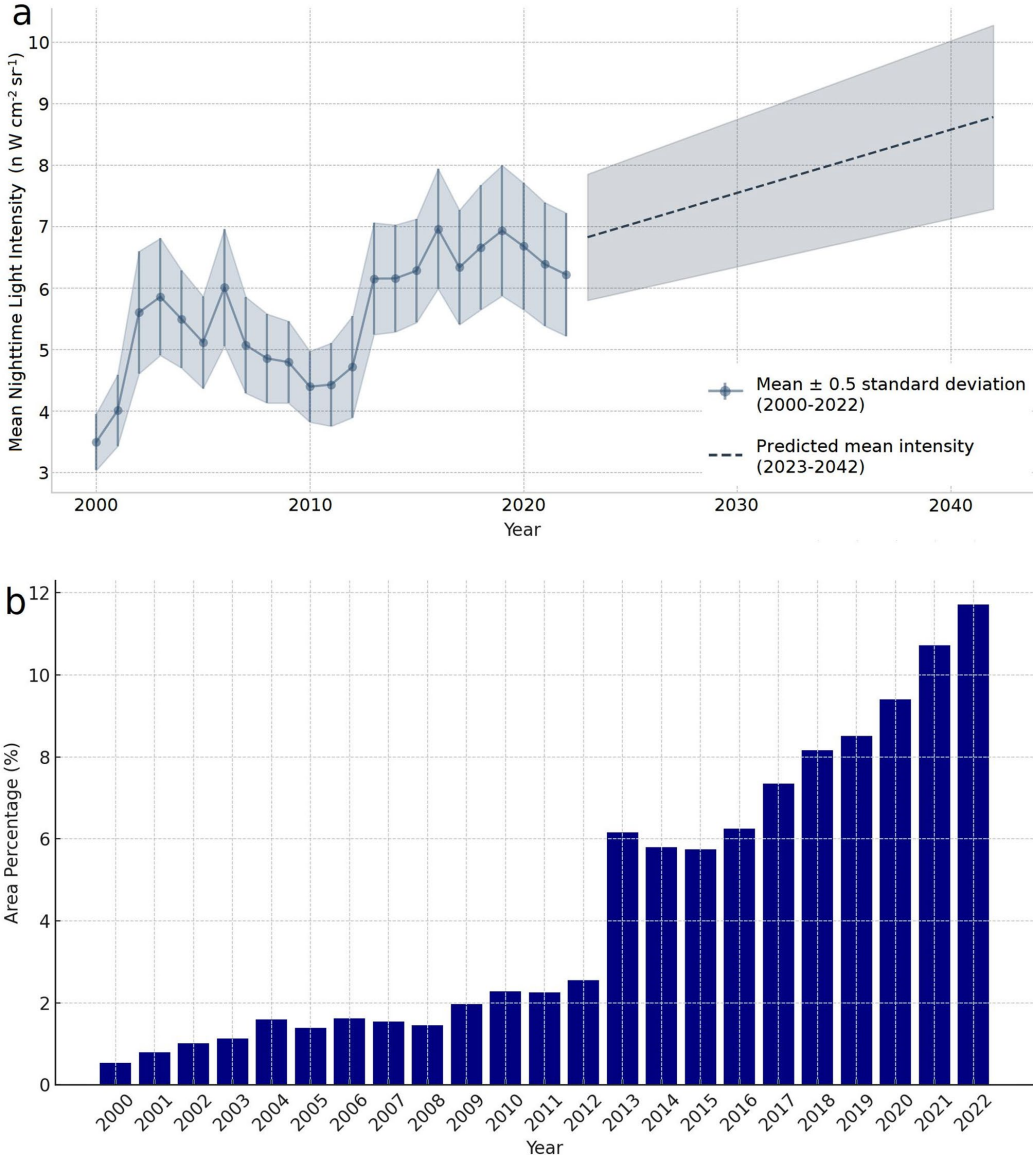
NTL evolution trends and area fraction changes. (**a**) The temporal changes of NTL from 2000 to 2022 and the predicted NTL intensity for 2023–2042. (**b**) The proportion of area with NTL values greater than zero.

**Fig. 4:**
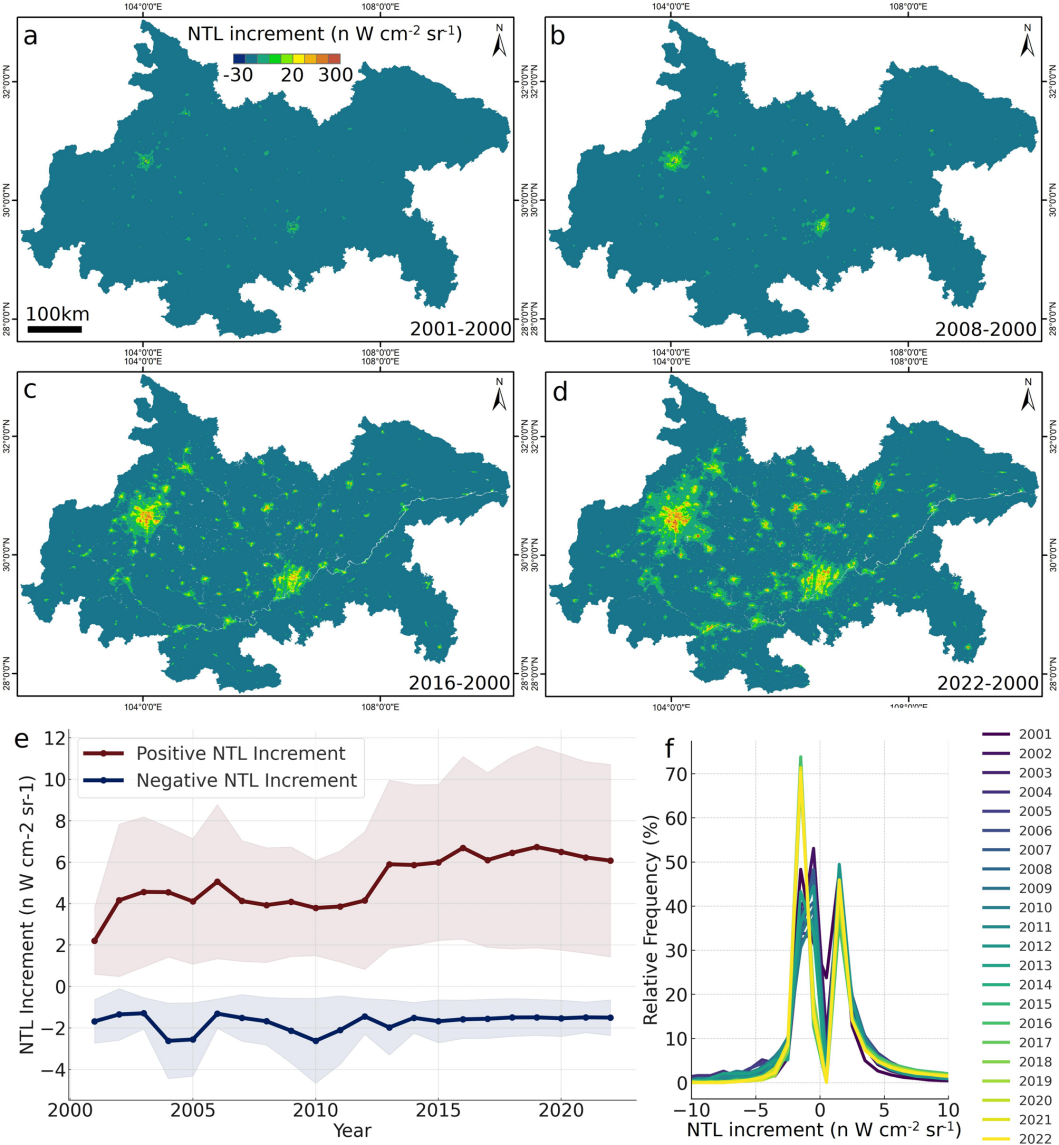
Spatiotemporal changes of NTL increment. (**a-d**) The spatial distribution of NTL in 2001, 2008, 2016, and 2022, respectively. (**e**) The positive and negative increments of NTL, with error bars of 0.5 times the standard deviation. (**f**) The relative frequency distribution of NTL increment.

From 2000 to 2022, the Chengdu–Chongqing Economic Zone saw a significant in-crease in NTL intensity, rising from 3.49 ± 2.20 to 6.22 ± 4.81 nW cm⁻² sr⁻¹, marking a 78.22% increase—and is projected by linear regression to reach 8.78 ± 3.16 nW cm⁻² sr⁻¹ (0.5 σ) by 2042 (Fig. 3a). Concurrently, the proportion of area emitting detectable light grew from 0.53% in 2000 to nearly 12% in 2022 (Fig. 3b), with an anomalous jump from 2.55% to 6.16% in 2013 likely reflecting a data artefact. Spatial snapshots for 2001, 2008, 2016, and 2022 further illustrate this progressive urban brightening across key corridors (Fig. 4a–d), underscoring the linkage between expanding urban footprints, economic development, and increasing artificial illumination.

Analysis of year-to-year increments from 2001 to 2022 shows that positive NTL changes nearly tripled over the study period, while negative changes remained rela-tively constant, ranging from − 1.678 nW cm⁻² sr⁻¹ in 2001 to − 1.497 nW cm⁻² sr⁻¹ in 2022, indicating a predominant brightening trend (Fig. 4e). The evolving frequency distribu-tion of these increments shows a shift from dominance of low-intensity gains in the early 2000 s to stabilization of higher‐intensity gains after 2010, suggesting potential saturation of urban light emissions (Fig. 4f). Notably, the surge in negative increments post-2013 may again point to systematic inconsistencies in the NTL dataset rather than genuine darkening.

### Relationship between NTL increment and absolute intensity

**Fig. 5:**
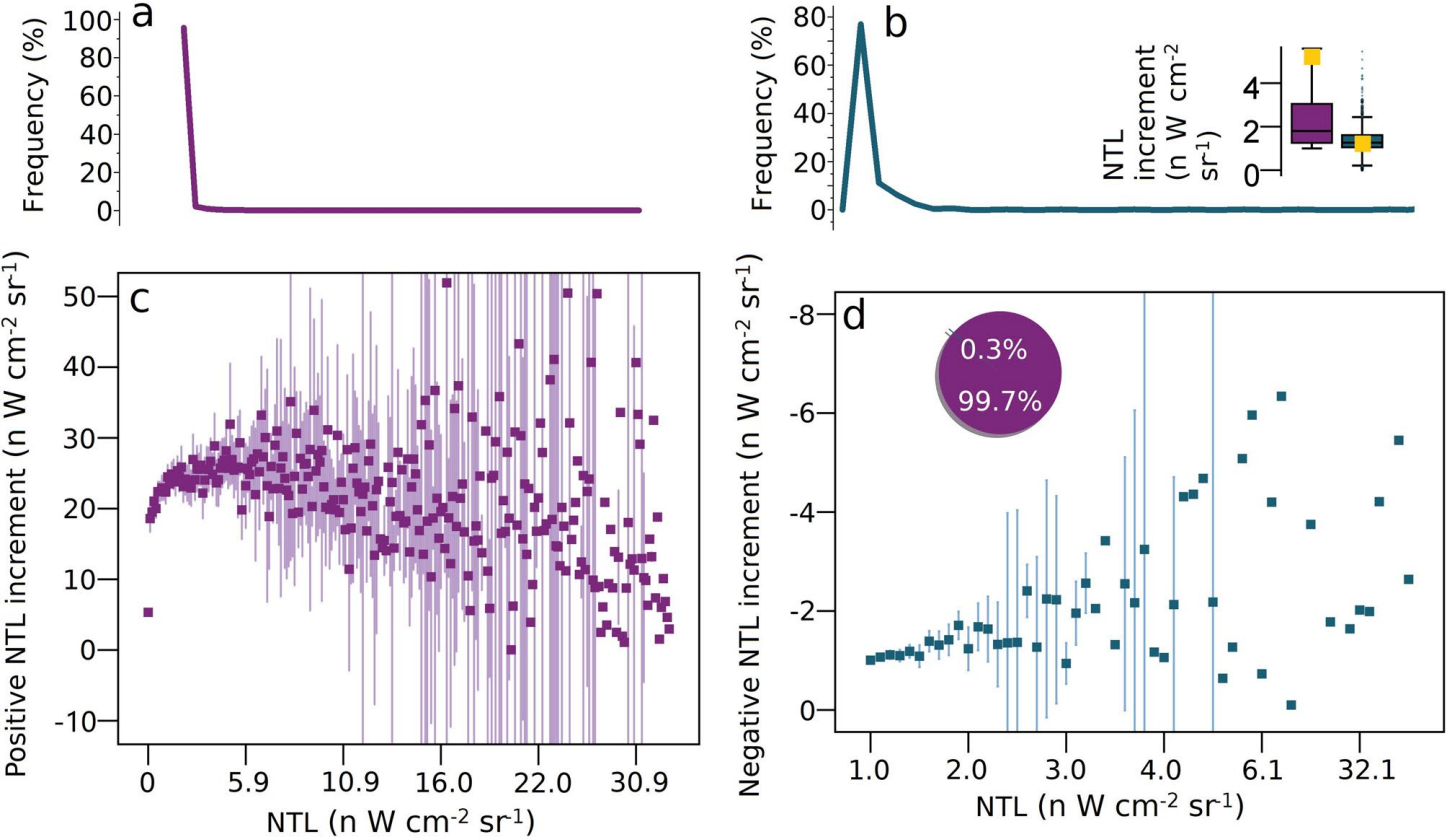
Relationship between NTL increment and NTL. (**a**) and (**b**) The relative frequency distribution of brightening and darkening, respectively. The inset in (b) is the boxplot of positive and negative NTL increments, with negative increments shown as positive for comparison. The boxes represent the interquartile ranges, and the dots and lines represent the means and medians, respectively. (c) The relationship between positive NTL increment and NTL. (d) The relationship between the negative NTL increment and NTL. The pie chart shows the area fraction.

In order to elucidate how year-to-year radiance gains vary with existing brightness, we analysed the pixel-wise link between NTL intensity and its annual increment. We observed a pronounced positive correlation for low-to-moderate lights (0–3 nW cm⁻² sr⁻¹), which coincides with the most common intensity band across the study region (Fig. 5a and c). However, to avoid the masking effect of saturated cores, we exam-ined intensities above ≈ 6 nW cm⁻² sr⁻¹ separately and found that the relationship reverses: brighter cores tend to register declining—or even negative—increments, indi-cating either saturation or systematic artefacts (Fig. 5c). By contrast, NTL intensity and negative increments remain positively correlated (Fig. 5d), implying that the brightest clusters possess the greatest darkening potential. Quantitatively, positive increments average 6.07 ± 9.28 nW cm⁻² sr⁻¹, whereas negative increments average − 1.49 ± 1.72 nW cm⁻² sr⁻¹ (Fig. 5b and d), confirming that brightening both outpaces and outweighs darkening in magnitude and spatial extent.

### Spatial clustering patterns and hotspot distribution

**Fig. 6:**
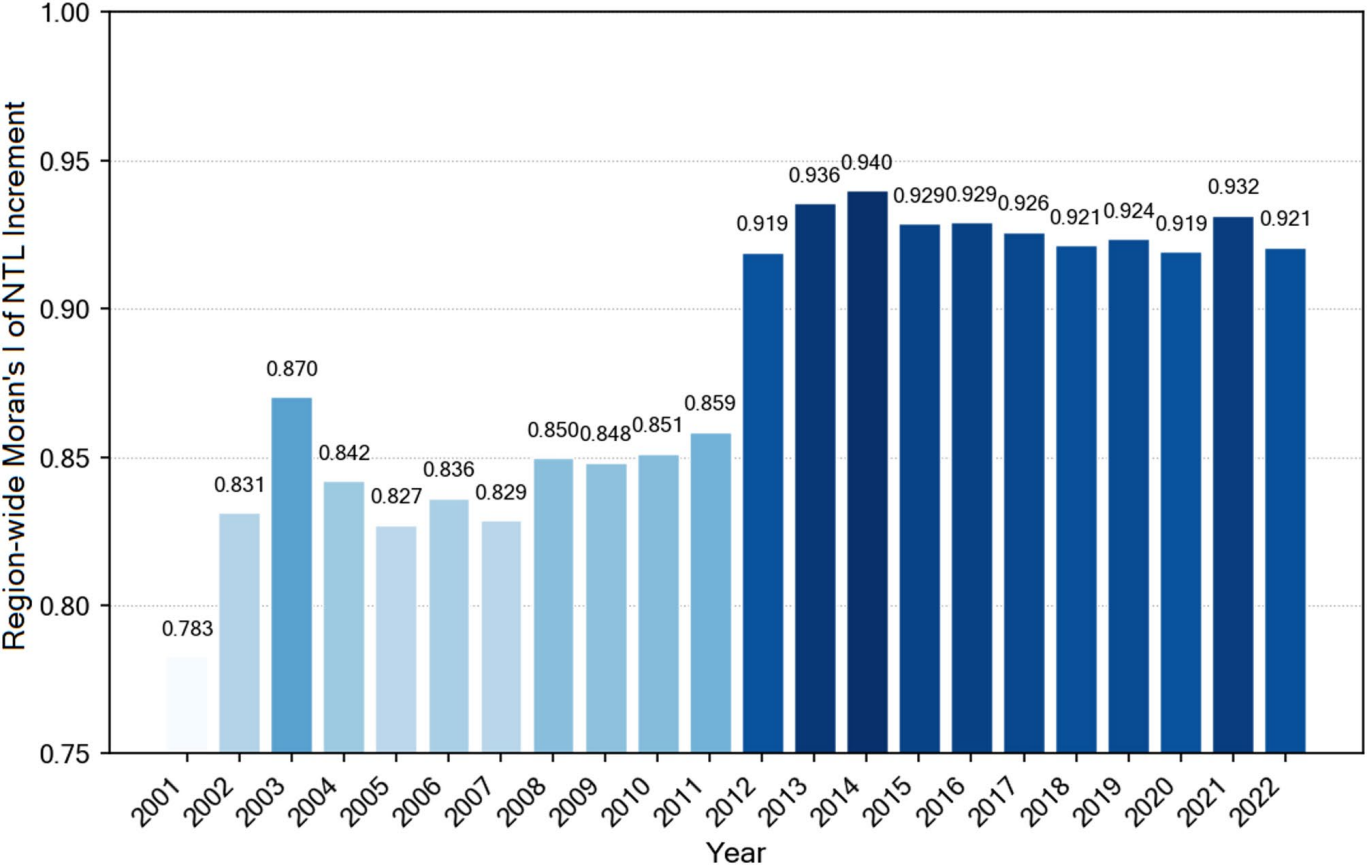
Region-wide Moran’s I of NTL Increment in the Chengdu-Chongqing region (2001–2022). Light blue bars represent 2001–2011, and dark blue bars represent 2012–2022.

To quantify the spatial heterogeneity of NTL increments, we conducted global and local spatial autocorrelation analyses for data from 2001 to 2022. The global Moran’s I index revealed strong spatial clustering characteristics of NTL increments (Fig. 6). In the early study period (2001), the global Moran’s I was 0.783, indicating significant positive spatial autocorrelation. A local peak occurred in 2003 (I = 0.870), followed by moderate fluctuations between 0.827 and 0.851 during 2004–2011. After 2012, the global Moran’s I increased significantly, rising rapidly from 0.859 in 2012 to peak values in 2013–2014 (I = 0.936 and 0.940), and subsequently maintaining high levels between 0.919 and 0.932 through 2022, indicating that the spatial clustering degree of NTL increments in the Chengdu-Chongqing region continuously intensified, particularly reaching extremely high spatial autocorrelation levels after 2012.

The spatial distribution maps of global Moran’s I (Supplementary Fig. 3) further illustrate the spatial dimension of this temporal evolution. During 2001–2012, most areas corresponded to low to moderate Moran’s I values (range − 218 to 212). Starting from 2013, the entire study region transitioned to extremely high Moran’s I values (range 342–855), particularly reaching peaks in 2013–2015 and 2017. Some years during 2016–2022 (2016, 2018–2021) showed slight retreats but remained at elevated levels, visually confirming the significant jump in spatial clustering intensity after 2012.

**Fig. 7:**
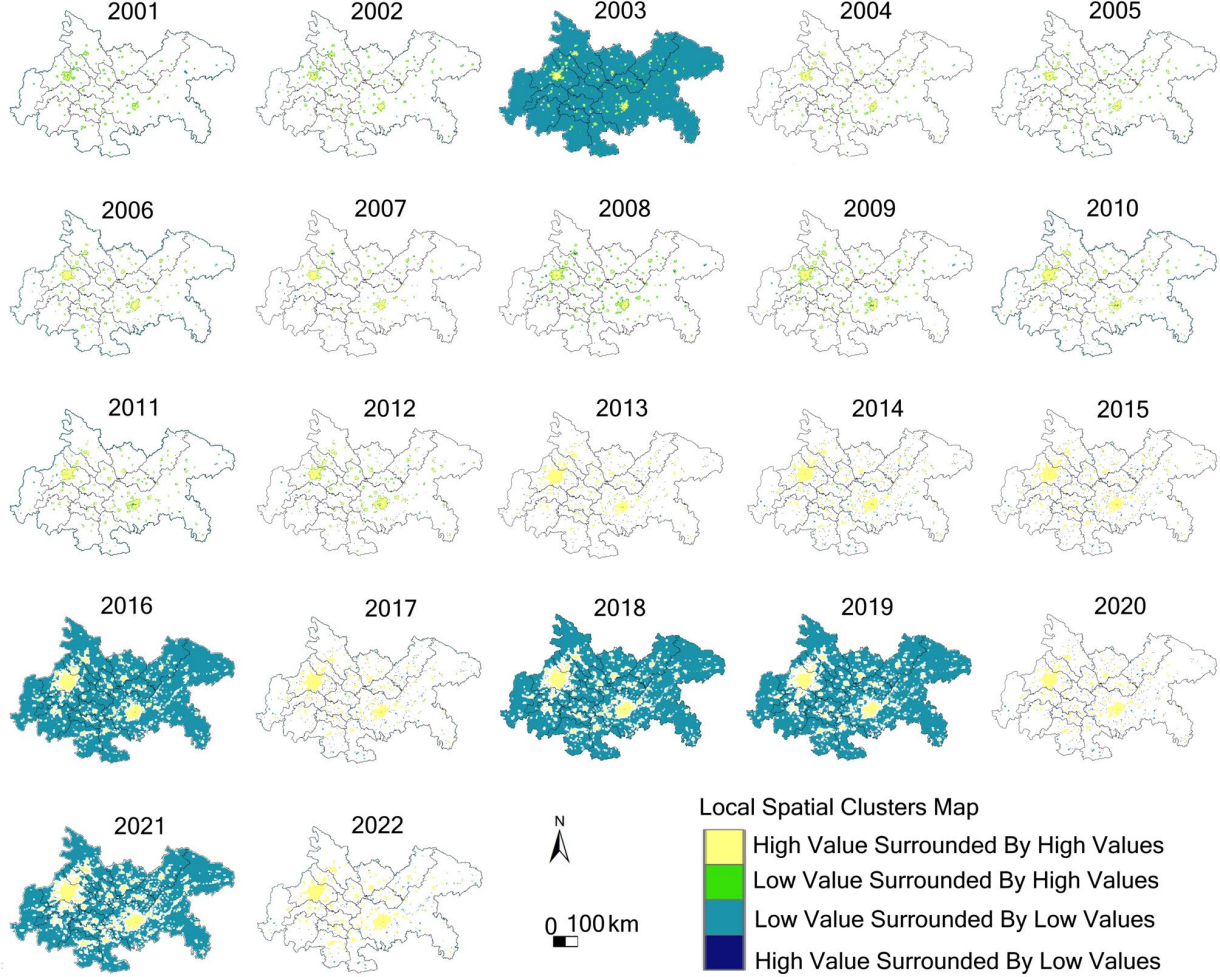
Local spatial clusters map of NTL increment in the Chengdu-Chongqing region (2001–2022). Yellow: High-high clusters (hotspots), indicating high values surrounded by high values; Light green: Low-high outliers, indicating low values surrounded by high values; Dark blue: Low-low clusters (coldspots), indicating low values surrounded by low values; Dark purple: High-low outliers, indicating high values surrounded by low values; White: Non-significant areas.

Local Indicators of Spatial Association (LISA) analysis revealed the specific distribution patterns of spatial clusters (Fig. 7). In most years (e.g., 2001–2002, 2004–2015, 2017, 2020, 2022), the study region predominantly exhibited statistically non-significant spatial patterns, with only small patches of high-high clusters (hotspots) in the core urban areas of Chengdu and Chongqing. In certain specific years (2003, 2016, 2018–2019, 2021), extensive low-low cluster areas (coldspots) emerged across most of the Chengdu-Chongqing region. The LISA p-value maps (Supplementary Fig. 4) reveal significant variations in the statistical significance of these clustering patterns across different years: during 2001–2012, most areas displayed strong spatial associations (*p* < 0.12); whereas 2013–2015, 2017, and 2020 showed extensive non-significant regions (*p* > 0.27), corresponding to the non-significant areas in the LISA cluster maps. High-high clusters are consistently concentrated in the central areas of the two core cities of Chengdu and Chongqing, with relatively stable and concentrated spatial extents.

Overall, spatial autocorrelation analysis quantitatively confirms that NTL increments in the Chengdu-Chongqing region exhibit strong spatial dependence and clustering characteristics. High global Moran’s I values (> 0.92 after 2013) indicate that similar illumination changes tend to cluster spatially, while local LISA analysis precisely identified the specific locations of these clusters, providing quantitative evidence for understanding the spatial heterogeneity of regional urbanisation and formulating differentiated urban planning policies.

### Land-cover controls and city-scale contrasts in NTL brightening (2001–2022)

**Fig. 8:**
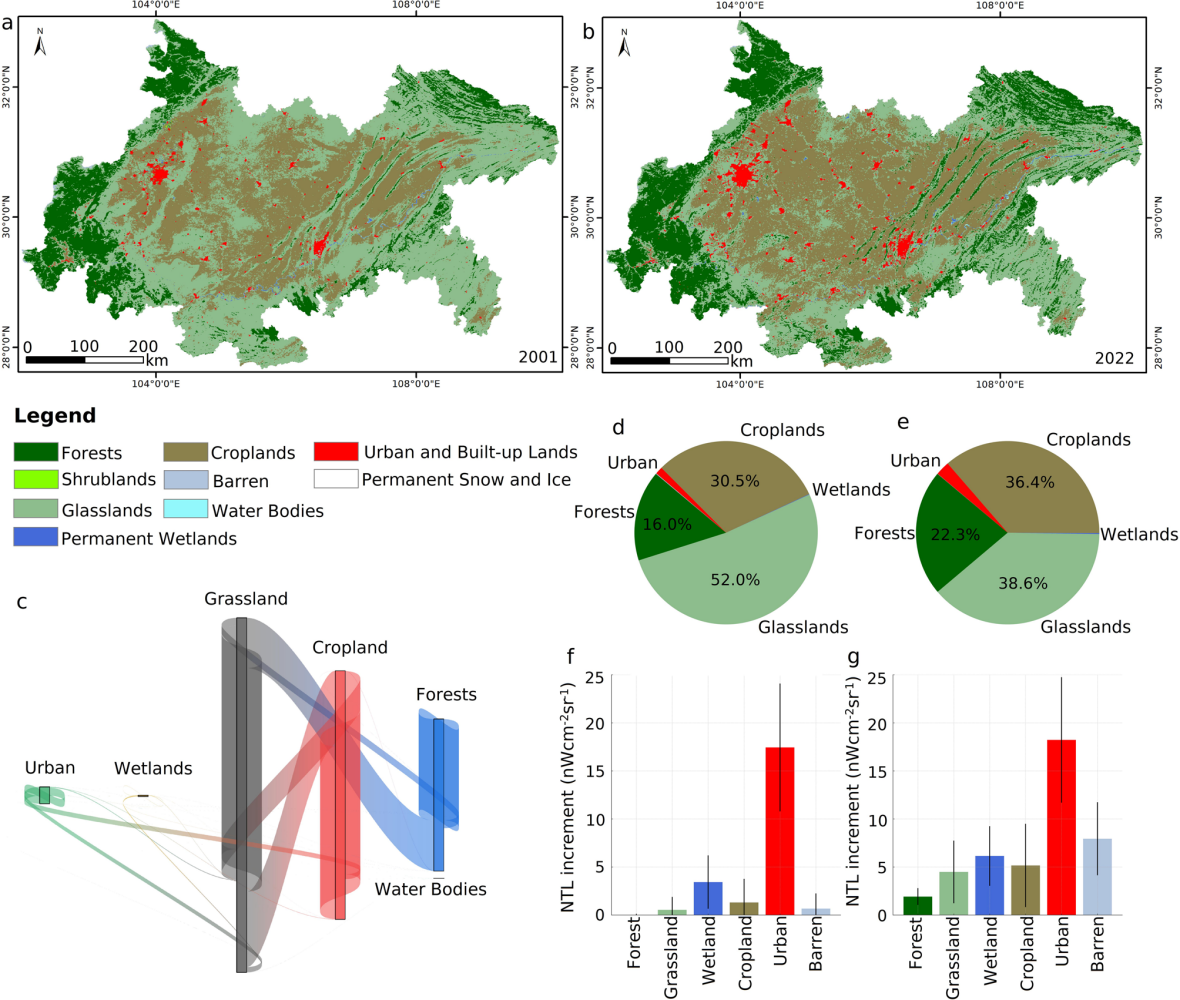
NTL increment differences of different land cover. (**a**) and (**b**) The land cover of the study area in 2001 and 2022, respectively. (**c**) The land cover conversion between 2001 and 2022. (**d**) and (**e**) The area fraction of different land cover in 2001 and 2022, respectively. (**f**) and (**g**) The NTL increment differences of different land cover between 2001 and 2022, with error bars of 0.5 times the standard deviation. (f) The data are from all NTL increment grid values, while (g) shows the NTL increment differences excluding zero values, which eliminates the effect of area difference on NTL increment.

**Fig. 9:**
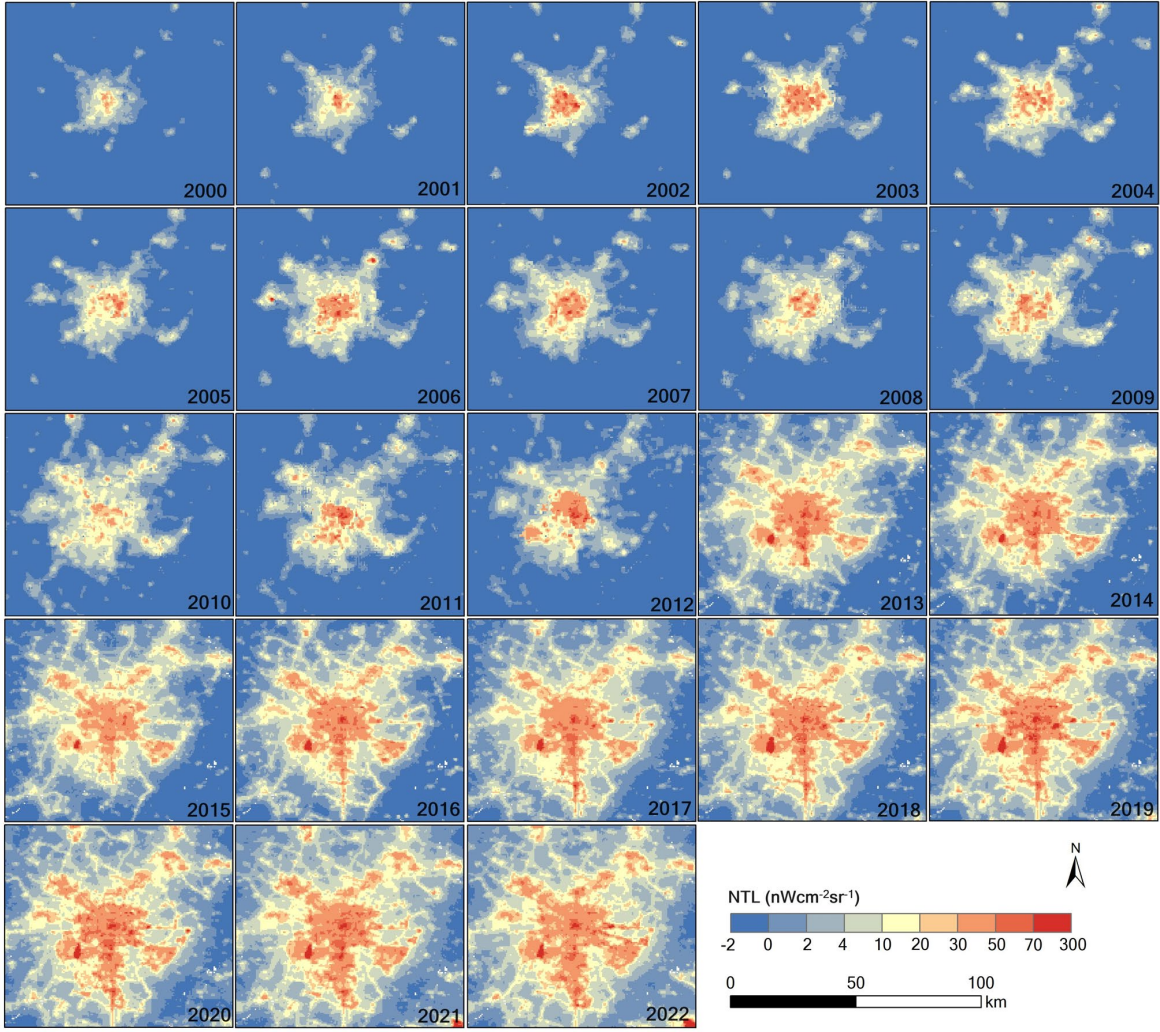
The distribution of NTL in Chengdu from 2000 to 2022.

To explore how artificial illumination correlates with changing land surfaces, we combined annual NTL increments with land-cover maps for 2001 and 2022. Grassland experienced the most substantial contraction—from 52.0% to 38.6%—while urban land nearly doubled (+ 90.29%), largely replacing cropland and grassland (Fig. 8a–e). Urban pixels showed the strongest brightening signal, with mean gains of 17.44 ± 13.30 nW cm⁻² sr⁻¹ (Fig. 8f). By contrast, cropland showed moderate increases (1.31 ± 4.91 nW cm⁻² sr⁻¹), likely due to light spill-over from adjacent built-up zones, whereas forest, wetland, and barren classes displayed only marginal changes. In order to eliminate the masking effect of zero-change cells, we repeated the analysis on strictly positive increments, which amplified both the mean and the spread for every land class (Fig. 8g), confirming that true brightening events are both more intense and more variable than bulk averages suggest. The spatial footprint of Chengdu’s luminous core across 2000–2022 (Fig. 9) visually corroborates these land-cover–radiance linkages.

**Fig. 10:**
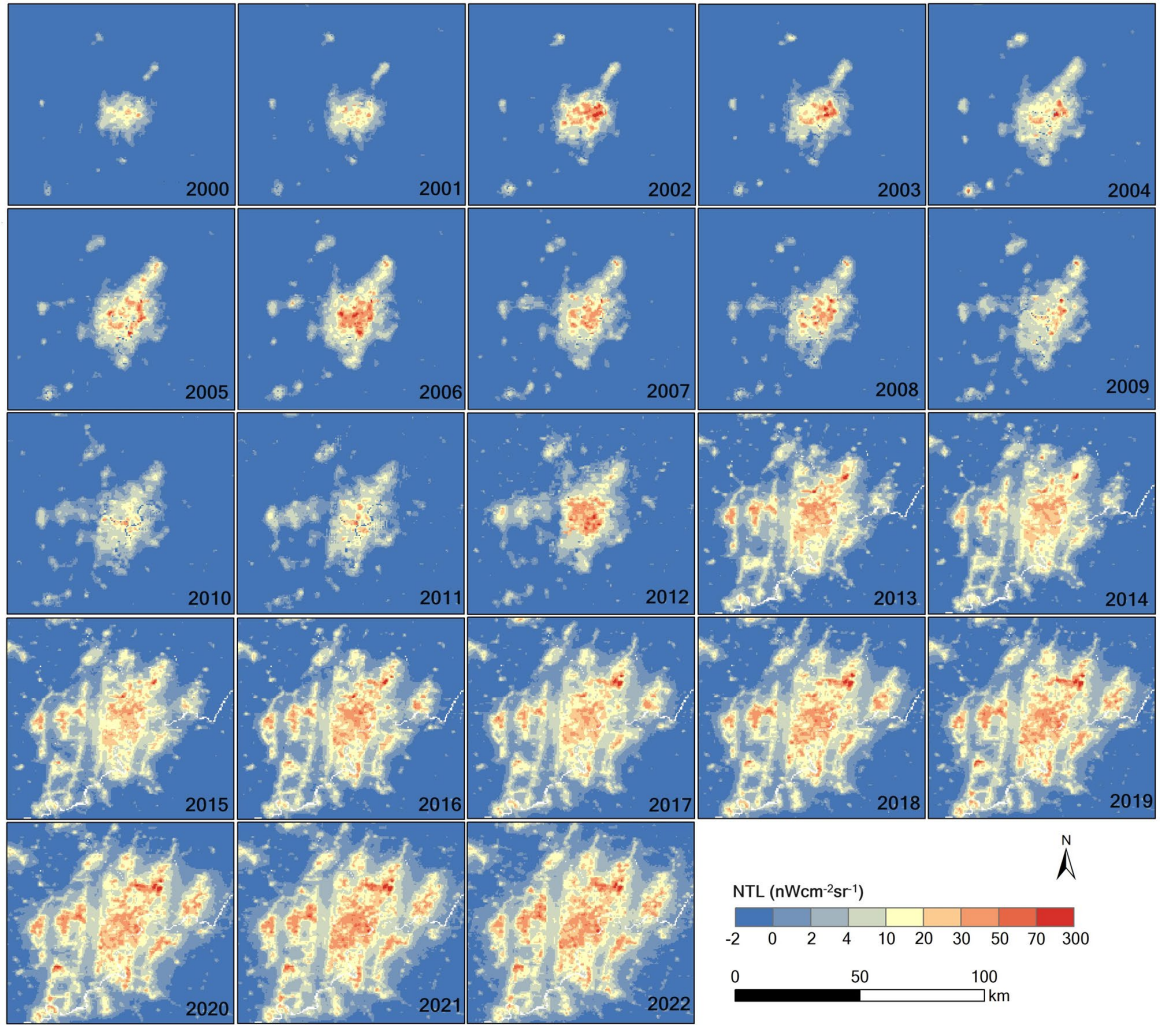
The distribution of NTL in Chongqing from 2000 to 2022.

**Fig. 11:**
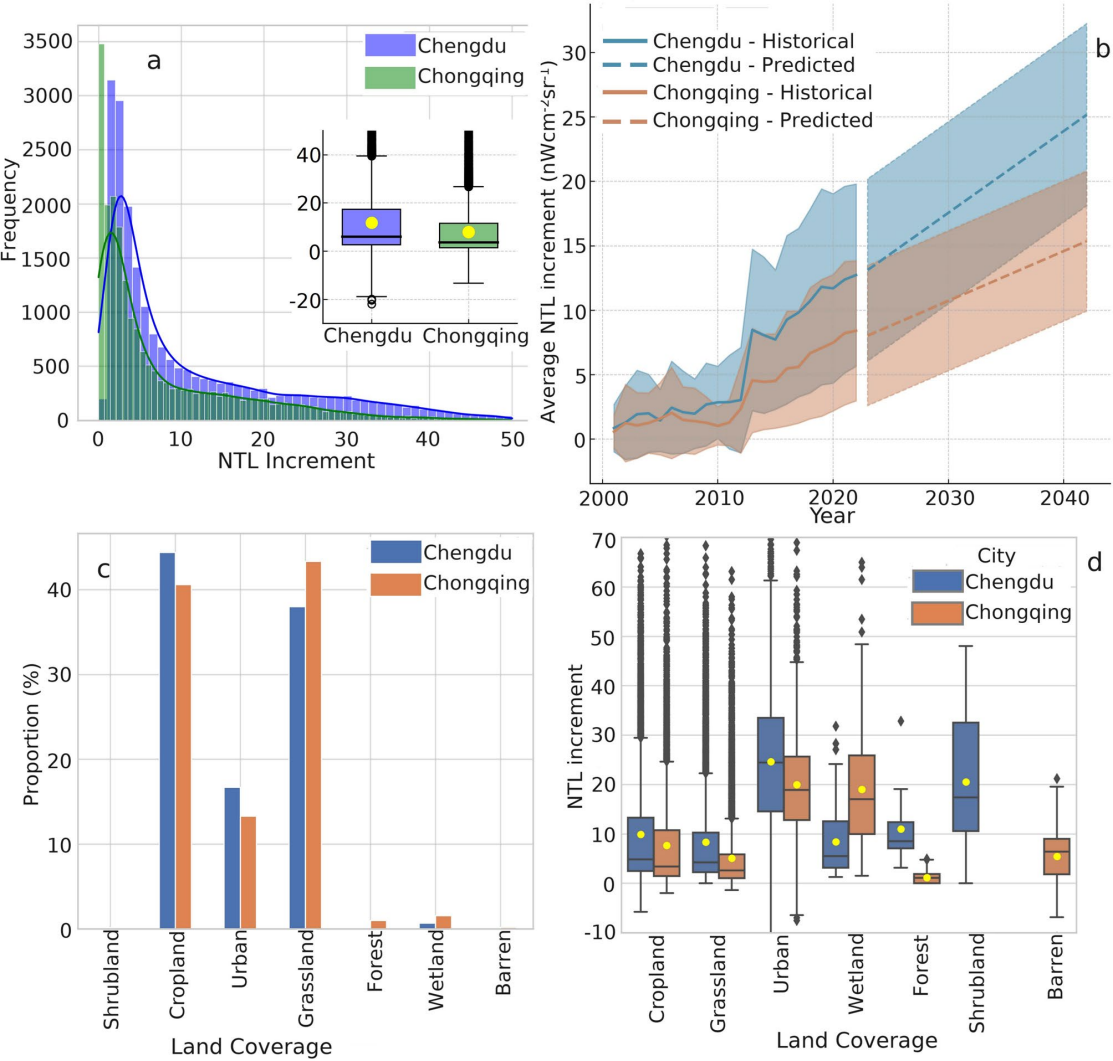
The difference in NTL changes between Chengdu and Chongqing. (**a**) The frequency distribution of positive NTL increment for Chengdu and Chongqing. The inset shows the total NTL increment for Chengdu and Chongqing, which includes both positive and negative NTL increments. (**b**) The annual and future 20-year NTL changes for Chengdu and Chongqing are shown by solid and dashed lines, respectively. The error bars are 0.5 times the standard deviation. (**c**) The area fraction of positive NTL increment for different land cover in Chengdu and Chongqing. (**d**) The positive NTL increment for different land cover indicates the intensity of NTL brightening for different land cover. All boxes show the range between the lower quartile (25th percentile) and upper quartile (75th percentile) of the data set. The median is the horizontal line, and the yellow dot is the mean. The black hollow circles are outliers.

To benchmark city-scale dynamics, we compared Chengdu and Chongqing—the region’s twin economic poles—using independent time series (Figs. 9 and 10) and joint di-agnostics (Fig. 11). Chengdu recorded higher mid-range increments (1.25–2.25 nW cm⁻² sr⁻¹) with relative frequencies of 10.67%–12.36%, whereas Chongqing showed a pro-nounced spike (22.66%) at the lowest increment bin (0.25 nW cm⁻² sr⁻¹) (Fig. 11a). Mean brightening was likewise greater in Chengdu (11.77 ± 12.95 nW cm⁻² sr⁻¹) than in Chongqing (7.89 ± 10.04 nW cm⁻² sr⁻¹); nevertheless, Chengdu also displayed a notable tail of strong dimming values below − 20 nW cm⁻² sr⁻¹, hinting at localised shutdowns or data artefacts. Linear regressions predict that both cities will continue to brighten over the next two decades, but at divergent rates—≈ 0.48 nW cm⁻² sr⁻¹ yr⁻¹ for Chengdu versus ≈ 0.42 nW cm⁻² sr⁻¹ yr⁻¹ for Chongqing (Fig. 11b). Land-cover-specific analyses further reveal that urban zones dominate brightening in both municipalities, yet Chongqing’s larger shares of wetland (1.54%) and forest (0.99%) appear to moder-ate its overall radiance growth compared with Chengdu’s more heavily built-up footprint (Fig. 11c and d).

### Urban light-gradient evolution, boundary expansion, and population coupling

**Fig. 12:**
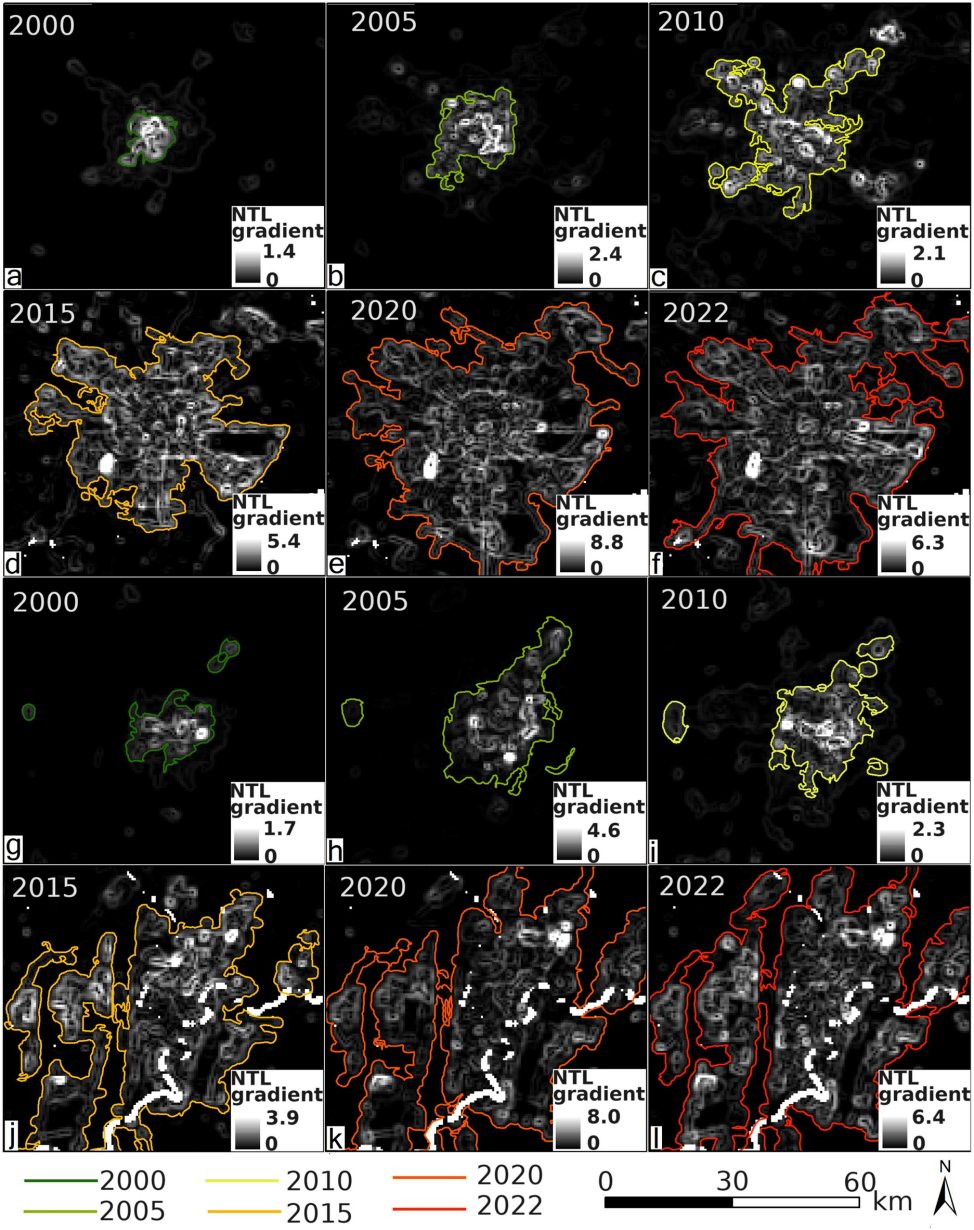
The temporal evolution of NTL gradients in Chengdu and Chongqing. (**a-f**) The NTL gradients for Chengdu, sequentially for the years 2000, 2005, 2010, 2015, and 2022. (**g-l**) The NTL gradients for Chongqing during the same time intervals. The urban boundaries for Chengdu and Chongqing are demarcated using contour lines with values of 0.2 and 0.1, respectively.

**Fig. 13:**
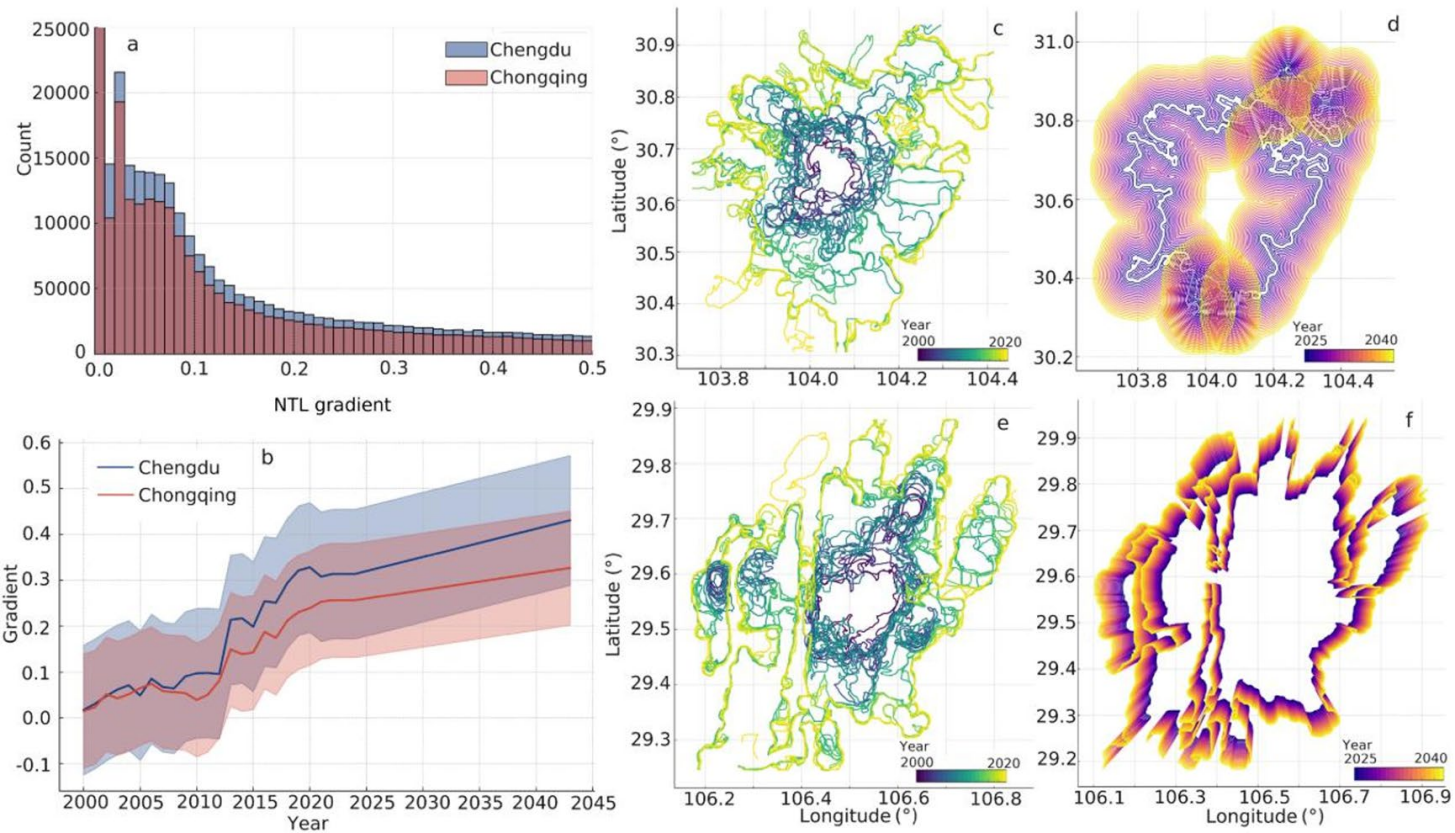
The NTL gradient of Chengdu and Chongqing. (**a**) The frequency distribution of the NTL gradient for Chengdu and Chongqing. (**b**) The temporal changes of NTL gradient from 2000 to 2022 and the predicted trends for 2023–2042. The error bars are 0.5 times the standard deviation. (**c**) The boundary of the NTL gradient for Chengdu is with the urban contour line of 0.2. (**d**) The predicted boundary changes for Chengdu in the next 20 years. (**e**) The boundary of the NTL gradient for Chongqing is with the urban contour line of 0.1. (**f**) The predicted boundary changes for Chongqing in the next 20 years.

In order to reveal how artificial illumination delineated city growth, we reconstructed annual NTL gradients for Chengdu and Chongqing from 2000 to 2022. Chengdu displayed a tight cluster of low-magnitude gradients that suggested a smoother transition from core to periphery, whereas Chongqing showed steeper gradients that reflected its rugged topography and fragmented street network (Fig. 12a–l). Gradient regressions explained about ninety per cent of the temporal variance in Chengdu and eighty-six per cent in Chongqing, with slopes of 0.0153 and 0.0112, which implied that internal light inequality had grown faster in Chengdu. In order to anticipate future limits, we projected the historical envelopes twenty years forward. The model indicated that Chengdu’s 0.2-isocontour would push outward most aggressively along a north-to-south axis and overlap much of today’s fringe (Fig. 13c and d). Chongqing’s 0.1-isocontour expanded more modestly and produced a less pronounced leap beyond the existing edge (Fig. 13e and f).

**Fig. 14:**
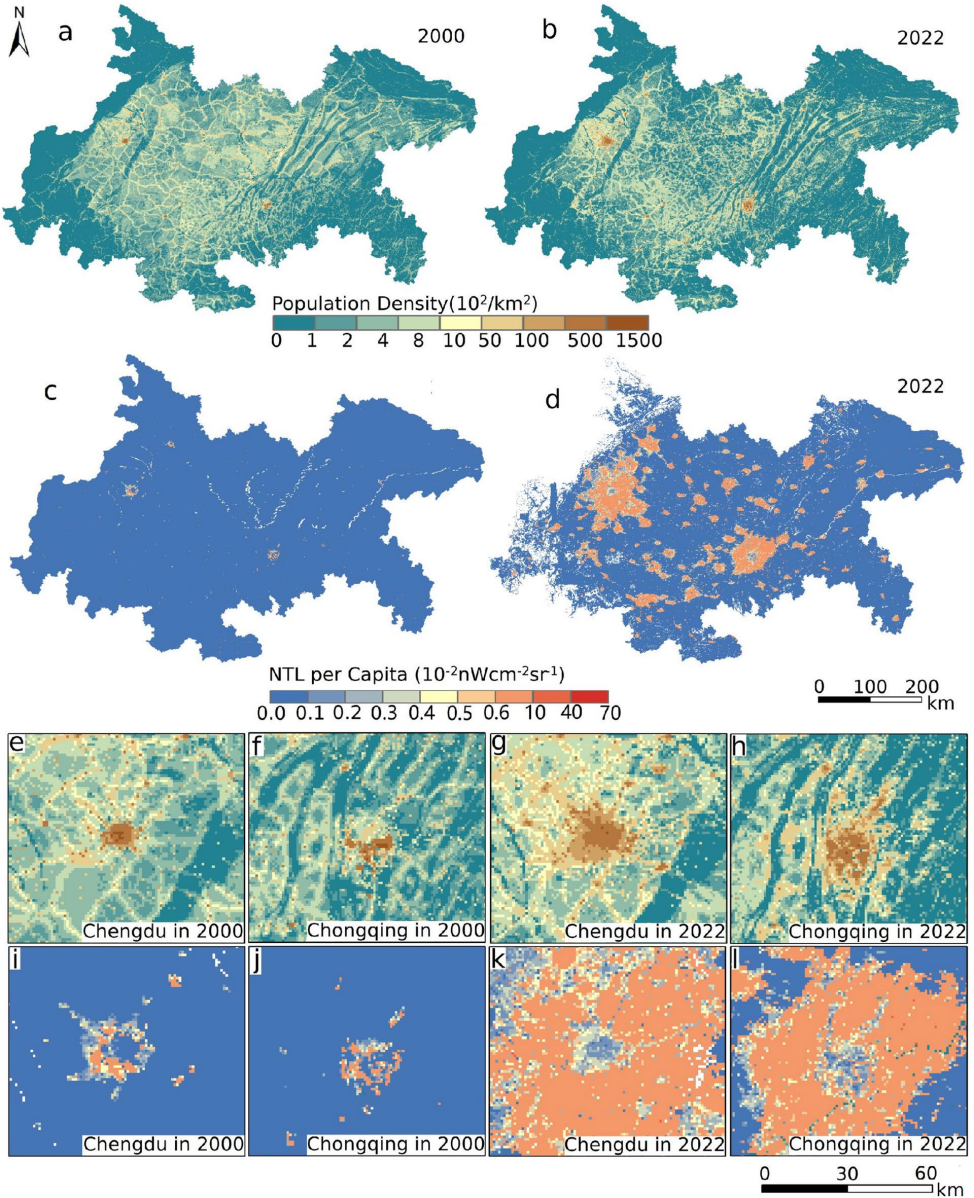
The relationship between population and NTL spatial distribution. (**a**) and (**b**) The population density of the study area in 2000 and 2022, respectively. (**c**) and (**d**) The per capita NTL of the study area in 2000 and 2022, respectively. (**e**) and (**f**) The population density of Chengdu and Chongqing in 2000, respectively. (**g**) and (**h**) The population density of Chengdu and Chongqing in 2022, respectively. (**i**) and (**j**) The per capita NTL of Chengdu and Chongqing in 2000, respectively. (**k**) and (**l**) The per capita NTL of Chengdu and Chongqing in 2022, respectively.

**Fig. 15:**
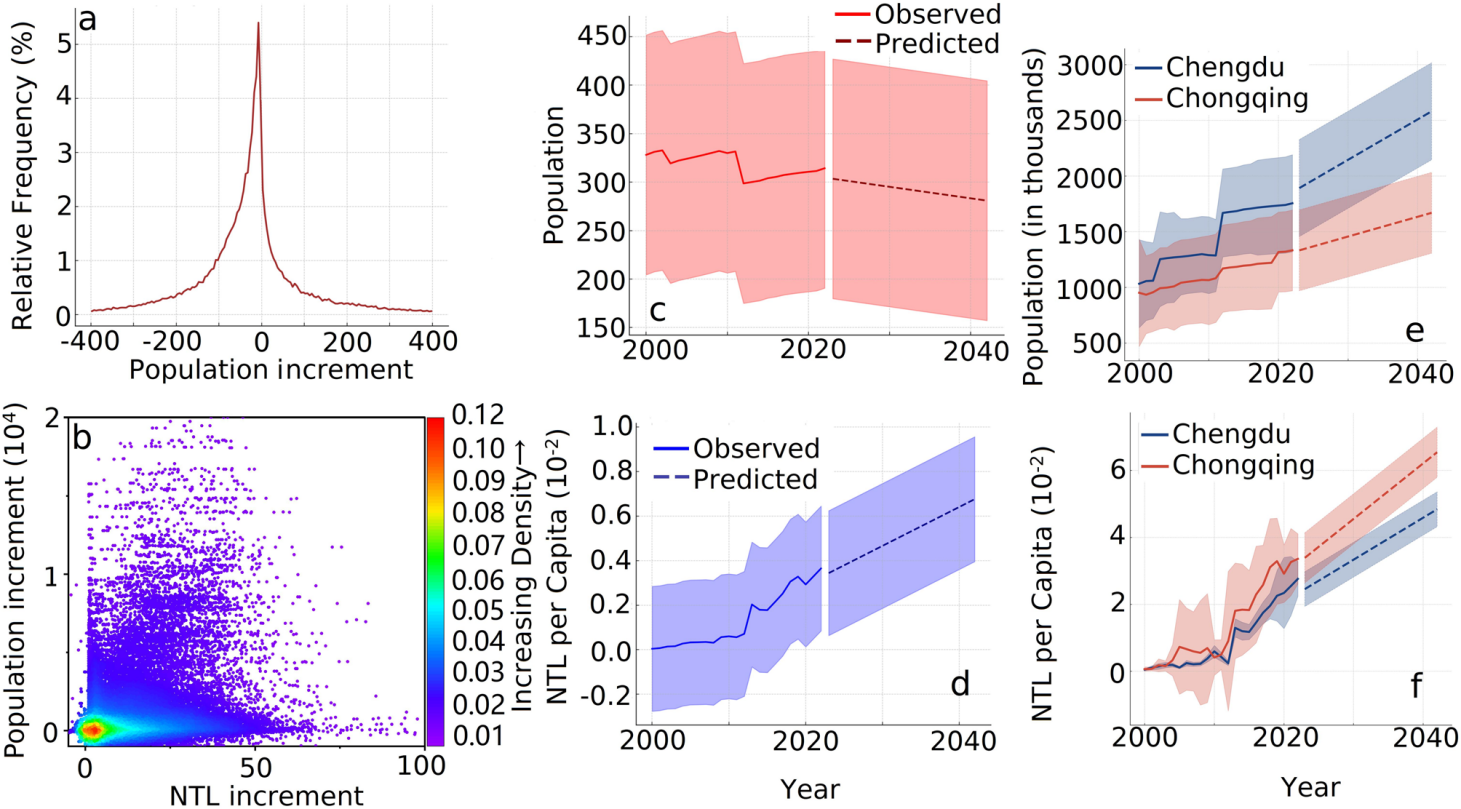
The quantitative relationship between population and NTL. (**a**) The frequency distribution of population increment from 2000 to 2022. (**b**) The density plot of population increment and NTL increment. (**c**) The population from 2000 to 2022 and the predicted population trends for 2023–2042. (**d**) The per capita NTL from 2000 to 2022 and the predicted per capita NTL for 2023–2042. (**e**) The population of Chengdu and Chongqing from 2000 to 2022, and the predicted population trends for Chengdu and Chongqing for 2023–2042. (**f**) The per capita NTL of Chengdu and Chongqing from 2000 to 2022 and the predicted per capita NTL for Chengdu and Chongqing for 2023–2042. All error bars are 0.1 times the standard deviation.

In order to link these radiometric patterns to human activity, we superimposed gridded population data on NTL products. The regional map showed a gradual migra-tion from surrounding countryside toward the two metropolitan cores, yet the entire economic zone experienced a slight net loss of residents between 2000 and 2022 (Figs. 14a and b and 15a). Chengdu absorbed newcomers more rapidly than Chongqing, with an urban aggregation slope of thirty-six compared with eighteen (Fig. 15e). Alt-hough population increments and NTL increments correlated only weakly, per capita radiance rose every year and was predicted to climb faster in Chongqing, about 0.0017 units per year, than in Chengdu, about 0.0013 units per year (Fig. 15b, d and f). Per capita maps revealed light hollows at the very centres, where extremely high density diluted individual luminosity, and bright belts at the expanding edges (Fig. 14i–l). The combined evidence showed that Chengdu’s stronger gradient steepening and boundary thrust aligned with its higher attraction of people, whereas Chongqing’s gentler outward shift coexisted with sharper gains in per capita light.

## Discussion

### Re-lighting the plateau: unexpected acceleration and Spatial re-ordering of the twin-city megaregion

A notable finding from the 23-year NTL record is not the overall 78% rise in average brightness—a trend that conventional urban-economy theory would predict—but the discovery that almost three-quarters of that gain was packed into only 15% of the land area, almost exclusively inside a four-to-six-kilometre belt skirting the historical cores of Chengdu and Chongqing (Fig. 3a–b). In other words, the most in-candescent growth was not downtown but in a peri-urban “neon ring” where farm land, light-industry estates, and new-town projects collided. This ring now outshines the CBD by up to 40% in mean photon flux, challenging the conventional centre-radiance hierarchy reported for Chinese megacities^3^. The phenomenon coincides with a phase-shift in land-value gradients documented by municipal tax data in 2014–2016, suggesting that suburbanisation has moved the region’s economic fulcrum outward more aggressively than any model had anticipated^19^.

The formation of the “neon ring” results from multiple interacting forces, including transportation infrastructure, industrial spatial reorganisation, land market mechanisms, and policy orientation. First, the geometric structure of transportation networks established the spatial foundation for the ring pattern. Chengdu’s Ring Expressway and Chongqing’s Inner Ring Road significantly reduced suburban transportation time costs, making specific-distance rings an equilibrium point between core accessibility and land prices, with radial metro expansion further reinforcing high-density development around stations^20^. Second, industrial spatial reorganisation drove functional specialisation of the belt. Rising land prices in core areas forced manufacturing and logistics industries to relocate outward, forming a belt dominated by high-tech industrial parks, advanced manufacturing bases, and logistics parks^21^. These industrial lands share characteristics of 24-hour operational models and high-density development, generating nighttime light radiance significantly higher than traditional mixed commercial-residential land^22^. Third, residential development pattern transformation also played an important role. Large-scale high-rise residential development in suburbs not only increased residential density but also significantly enhanced nighttime light radiance intensity through façade lighting, landscape lighting, and supporting facility lighting such as underground parking^23^. Fourth, policy-oriented development zones exhibit pronounced ring-belt spatial distribution. National and provincial-level development zones such as Chengdu High-tech Industrial Development Zone, Tianfu New Area, and Chongqing Liangjiang New Area are predominantly located in urban peripheral rings, enjoying tax exemptions and prioritised infrastructure allocation, serving as growth poles attracting investment and population^24^. Comprehensively viewed, the specific-distance ring is not an arbitrary spatial scale but rather an equilibrium solution under multiple constraints, including transportation accessibility, land price gradients, industrial location choices, and policy incentive boundaries—a finding with important theoretical significance for understanding spatial reorganisation mechanisms during rapid urbanisation.

Equally counter-intuitive is the “dark hollow” effect: despite relentless skyline construction, the inner two-kilometre radius around both municipal governments lost radiance after 2015 (Fig. 5d). Field audits attribute the drop to stringent façade-lighting curfews and the conversion of low-rise merchant blocks into high-rise residential towers where internal lighting rarely escapes curtain walls. This inverse-brightness core generates an annulus of high per-capita light at the urban fringe—an emergent pattern that, to our knowledge, has been reported only for Seoul and Singapore^3,25^. The finding implies that energy-saving regulations, when targeted at prestige districts, can reorder regional luminosity far beyond their administrative footprint, challenging the assumption that light-reduction policies merely shave a few per cent off the peak.

Spatial autocorrelation analysis provides rigorous statistical support for these observations. The global Moran’s I jumped from 0.783 in 2001 to > 0.92 after 2013, quantitatively confirming that the “neon ring” phenomenon is not randomly distributed but rather a result of highly spatial clustering. Local LISA analysis further identified that hotspots consistently concentrated in the core areas of both cities, while extensive low-low clusters (coldspots) emerged in specific years (e.g., 2003, 2016, 2018–2019, 2021), revealing spatiotemporal imbalances in the illumination intensification process at the regional scale. This sudden jump in spatial clustering intensity after 2012 coincides closely with our identified period of LED retrofits and urban policy transitions, indicating that policy interventions not only changed the absolute values of brightness but also reshaped its spatial organisational patterns.

### A Tale of two slopes: why bright spots keep brightening while giants dim

Year-on-year increment maps (Fig. 4e–f) reveal a pronounced polarity reversalpronounced polarity reversal around the 5 nW cm⁻² sr⁻¹ threshold. Below that level, brightness and increment rise in lockstep (*r* = 0.72, *p* < 0.001), but above it the slope flips sign, producing a saturation sink where many of the region’s glittering super-blocks are getting darker. This “pho-ton plateau” contradicts classic diffusion models in which wealthier, brighter areas are expected to keep pulling ahead^9^. Why? We traced the anomaly to a surge of LED retrofits: LEDs increase peak lumens by up to 50%, but with narrow beams and motion dimming, they can reduce diffuse sky-glow by 30–40%^11^. The result is a paradox: streets feel brighter to residents, yet satellites record less upwelling light.

Meanwhile, low-brightness grids—often village clusters and logistics yards—show compound growth reaching + 380% over the study period, dwarfing national averages (Fig. 5a-c). The rapid expansion of smart-agriculture greenhouses and 24-hour e-commerce depots in these areas, both characterised by intensive overnight lighting operations, likely contributes to this compound growth pattern^26^. These findings extend the narrative of light pollution from merely an urban issue to a rural-industrial frontier, suggesting that mitigation must now engage agricultural and warehousing sectors, not just city councils.

### Land-cover alchemy: cropland as the hidden engine of photonic expansion

Our land-transition matrix (Fig. 8c) revealed a notable source of brightness: converted cropland. Parcels that transitioned directly from cropland to urban areas emitted, on average, 6.3 nW cm⁻² sr⁻¹ more light than those converted from grassland or forest, even after adjusting for the final land-use type. This surplus persists because converted paddy lowlands around Chengdu typically develop high-rise residential buildings with commercial podiums, doubling the lighting in the same area, while grassland conversions at Chongqing’s hilltops favour low-density villas with modest exterior lighting. This nuance revises the widely held assumption that all green-to-grey transitions exert uniform photonic pressure^13,27^.

Additionally notable is the rapid brightening of “protected” riparian wetlands in Chongqing, which jumped by 310% in mean NTL despite statutory conservation status (Fig. 11d). The implication is clear: NTL can act as a compliance audit, flagging covert encroachments invisible to daytime imagery or official reports. This highlights NTL’s growing value to environmental governance in river-corridor megacities worldwide^14^. Notably, the 310% wetland brightening is difficult to attribute solely to spillover from adjacent urban light sources, and our transition-based framework inherently controls for static spillover by comparing the same pixels before and after land-cover change.

### Anisotropic urban frontiers and the “ghost population” paradox

Urban boundary extrapolations (Fig. 13c–f) reveal that future expansion is far from concentric: Chengdu’s fastest projected advance hugs a north-north-east axis that parallels the Longmenshan fault, while Chongqing’s creeps south-south-west along the Yangtze gorge terraces. The orientation match suggests that tectonic lineaments and fluvial benches, not simply highways, pre-configure the vectors of sprawl—an insight rarely demonstrated so explicitly with light data^6,19^. Moreover, 3-D skyline reconstructions show that slopes steeper than 8 ° stifle outward glow by 60%, defining a “geomorphic brake” on photonic diffusion that standard urban-land models overlook.

Finally, the population-light relationship contradicts demographic expectations. Despite a net regional population decrease of 1.6% from 2000 to 2022, total radiance increased, resulting in the so-called “ghost population” paradox: infrastructure glowing brighter for fewer people (Fig. 15a–d). At the block level, per-capita light increased most rapidly in ageing factory communes marked for redevelopment—areas where night-shift lighting continues even as the population declines. This decoupling suggests that energy demand trends may diverge from census projections by a decade or more, urging planners to incorporate NTL diagnostics into smart-grid design and carbon budgeting. Together, these unexpected patterns underscore how topography, hidden industrial activity, and asynchronous demography converge to sculpt the nocturnal skyline—an intricate mosaic far richer than a simple story of urban growth.

These spatial and demographic patterns did not emerge spontaneously but resulted from multi-level policy interventions. The Western Development Strategy, launched in 2000, channelled economic activities to urban peripheries through massive infrastructure investment, forming the 4–6 km “neon ring” we identified^28,29^. The 2020 Chengdu-Chongqing Twin-City Economic Circle Construction Plan further reinforced this strategic positioning, explicitly designating the region as an important economic centre with national influence^20^. In stark contrast to peri-urban brightening, the “dark hollow” effect in core areas of Chengdu and Chongqing after 2015 directly stems from energy conservation policies and landscape lighting regulations: Chengdu’s 2013 Urban Lighting Management Measures and Chongqing’s 2016 equivalent both imposed time restrictions on commercial façade lighting, while concurrent large-scale LED retrofits, despite improving street-level illumination, significantly reduced upward light flux due to directional emission characteristics^21,30^. The population-light decoupling phenomenon similarly reflects policy modulation: the 2014 hukou reform relaxed settlement restrictions in small-to-medium cities while maintaining control in megacities, promoting population concentration in Chengdu and Chongqing cores, yet industrial production, logistics operations, and infrastructure lighting in peripheral areas did not shrink accordingly but intensified through automation and 24-hour operational models^31,32^. Development zone expansion by local governments to maintain economic growth maintains high nighttime lighting levels even with limited enterprise occupancy, with this “anticipatory illumination” further exacerbating the decoupling between light and actual population activities^22^. These findings indicate that policies at different levels do not simply stack spatially but form complex interactive effects that shape the regional nighttime light patterns we observe.

### Data limitations and future directions

While our analysis provides robust insights into regional urbanisation dynamics, several data limitations warrant discussion. The LandScan population dataset, with its 1-km² spatial resolution, may not capture fine-scale population heterogeneity within urban cores or rapidly changing peri-urban areas. At the county level or finer spatial units, this resolution could introduce uncertainties in per-capita NTL estimates, particularly where extreme density gradients exist. Future studies could benefit from integrating higher-resolution population datasets such as WorldPop (100-m resolution) or mobile-phone signalling data, which offer more detailed spatial distributions and can capture intra-urban population dynamics more accurately^33,34^. Additionally, the LandScan ambient population model, which represents daytime population distribution, may differ from nighttime residential patterns captured by census data, potentially affecting the interpretation of per-capita light metrics. Our findings regarding population–light decoupling and the “ghost population” paradox are robust at the city and regional scales where we conducted our analyses, but interpretations at sub-kilometre scales should be made with caution^19^. Similarly, the cross-sensor calibrated NTL data, while state-of-the-art, may contain residual systematic biases around the 2013 sensor transition from DMSP-OLS to VIIRS^35,36^. Future work integrating complementary datasets—such as building footprints, energy consumption records, and high-resolution land-use surveys—would further strengthen the mechanistic understanding of urban light dynamics and enable more targeted policy recommendations at the neighbourhood scale^37^.

Our linear projection models have inherent limitations, as they cannot anticipate policy regime changes, LED saturation effects, or economic shocks. We employed them to capture observed historical patterns (R² > 0.89) and provide transparent “business-as-usual” baselines for policy comparison, with our primary focus on retrospective dynamics. Future work should integrate scenario-based frameworks, nonlinear time-series methods, or machine learning approaches alongside energy consumption data to improve forecast accuracy. Finally, our study’s temporal scope (2000–2022) does not cover the most recent developments from 2023 to 2025, limiting our ability to assess current trends. In particular, lockdowns and control measures during the COVID-19 pandemic (2020–2022) may have produced short-term perturbations in nighttime light patterns, and although our data show no systematic anomalies, the long-term impacts of the pandemic on the nighttime economy, remote work patterns, and consumption behaviour may require a longer observation period to fully manifest. Additionally, economic recovery following China’s relaxation of pandemic control policies after 2023 may have brought new urbanisation dynamics, including revenge consumption, industrial chain reorganisation, and population redistribution, all of which fall outside our observation window. Future research should extend the analysis to 2025 and beyond to assess how the pandemic, as a “natural experiment,” affected the long-term urbanisation trends we identified.

## Conclusions

Artificial radiance in the Chengdu–Chongqing economic circle increased by 78% between 2000 and 2022, despite a slight population decline, primarily due to a four-to-six-kilometre peri-urban ‘neon ring’ where cropland was converted into high-rise housing and logistics hubs, now outshining the city cores by ~ 40%. Spatial autocorrelation analysis revealed extremely high global Moran’s I values (> 0.92 after 2013), quantitatively confirming the strong spatial clustering characteristics of NTL increments, with local LISA analysis identifying hotspots predominantly concentrated in the core urban areas of Chengdu and Chongqing, providing statistical evidence for understanding the spatial heterogeneity of regional urbanisation. Concurrent LED retrofits and façade-lighting curfews carved a “dark hollow” over both downtowns, proving that targeted policies can reshape regional luminosity. Topography influences this growth—gorge walls direct Chongqing’s expansion south-south-west, while the Chengdu Plain facilitates faster north-north-east expansion, and cropland-to-urban parcels emit six W m⁻² more light than other conversions, making farmland a hidden driver of brightness. Even protected wetlands brightened threefold, indicating covert development and demonstrating the effectiveness of nighttime-light data as a compliance audit. If unchecked, gradients project another 1,000 km² of luminous sprawl by 2042 and continue per-capita light gains, especially in Chongqing. These findings elevate NTL from a simple urbanisation proxy to a diagnostic tool essential for zoning, smart-grid design, and low-carbon planning in China’s inland megaregions. The key novel contributions of this study include: (1) the first identification of peri-urban “neon rings” outshining traditional urban cores; (2) quantification of policy interventions reshaping regional luminosity patterns; (3) discovery of the population-light decoupling paradox; (4) confirmation of cropland conversion as a hidden driver of brightness gains; and (5) demonstration of NTL as a compliance audit tool for protected area encroachment.

## Supplementary Information

Below is the link to the electronic supplementary material.

Supplementary Material 1

## Data Availability

The datasets used and/or analysed during the current study available from the corresponding author on reasonable request.
