# Supplementary material for: Nighttime light dynamics reveal peri-urban brightening and population decoupling in the Chengdu Chongqing megaregion
Source: Sci Rep. 2026 Jan 10;16:4601. doi: 10.1038/s41598-025-34706-9 (PMC12868891; doi:10.1038/s41598-025-34706-9)
Supplement: Supplementary file 1 — Supplementary Material 1 [file 41598_2025_34706_MOESM1_ESM.docx]

Supplementary Materials for

**Nighttime Light Dynamics Reveal Peri-urban Brightening and Population Decoupling in the Chengdu Chongqing Megaregion**


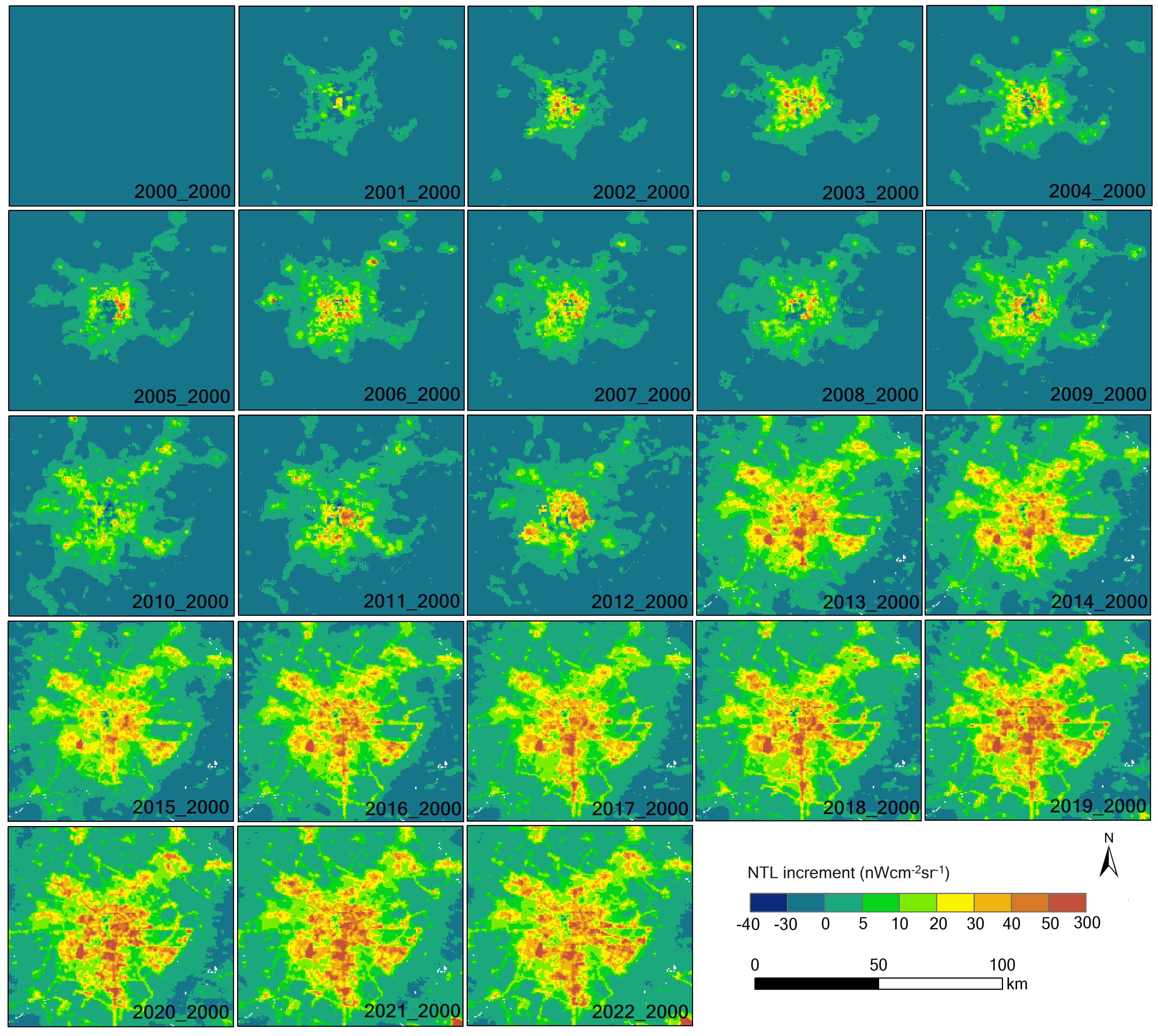
Supplementary Figure 1 The annual incremental changes in NTL intensity in Chengdu during the survey period


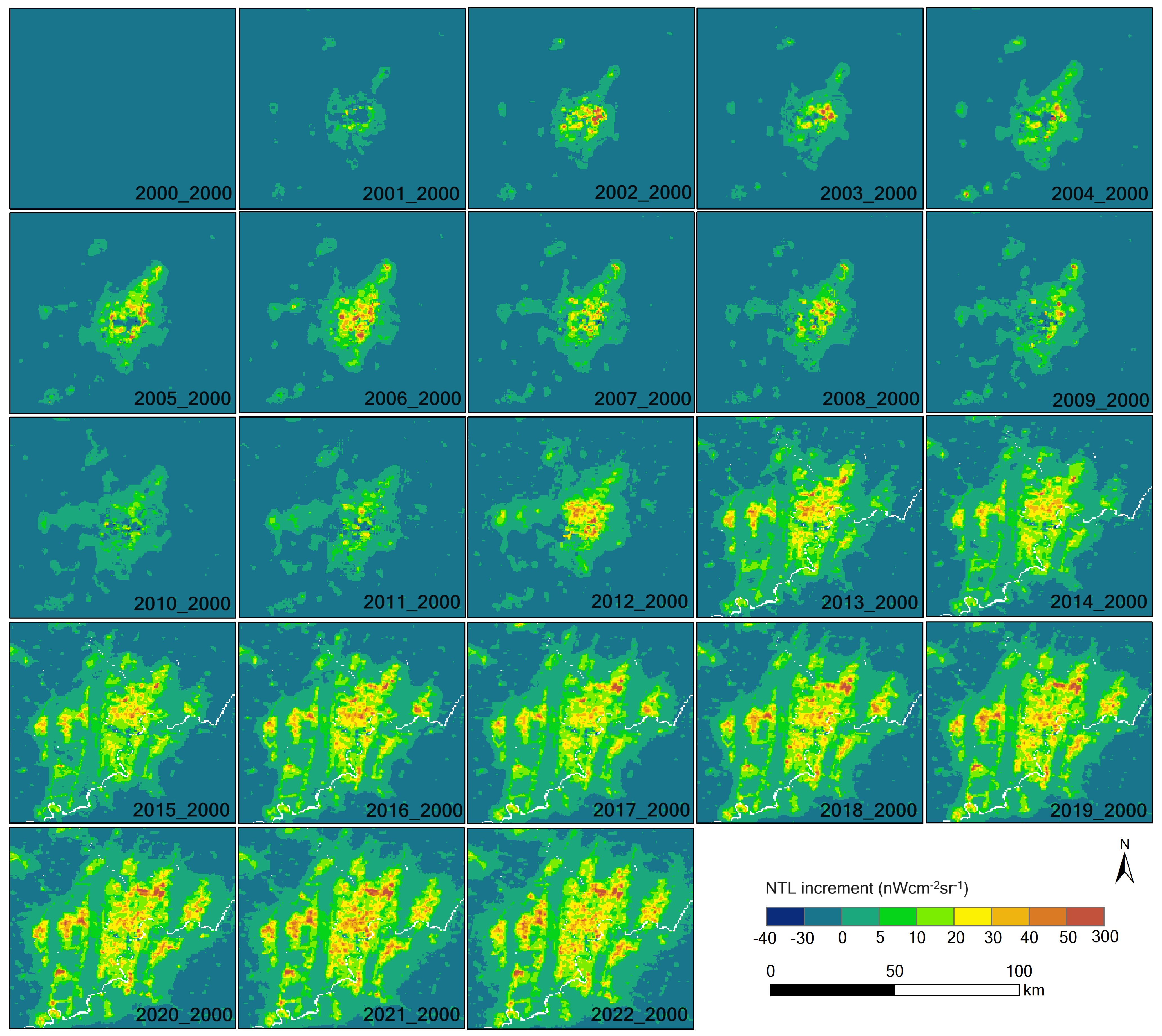
Supplementary Fig.2 The annual incremental changes in NTL intensity in Chongqing during the survey period


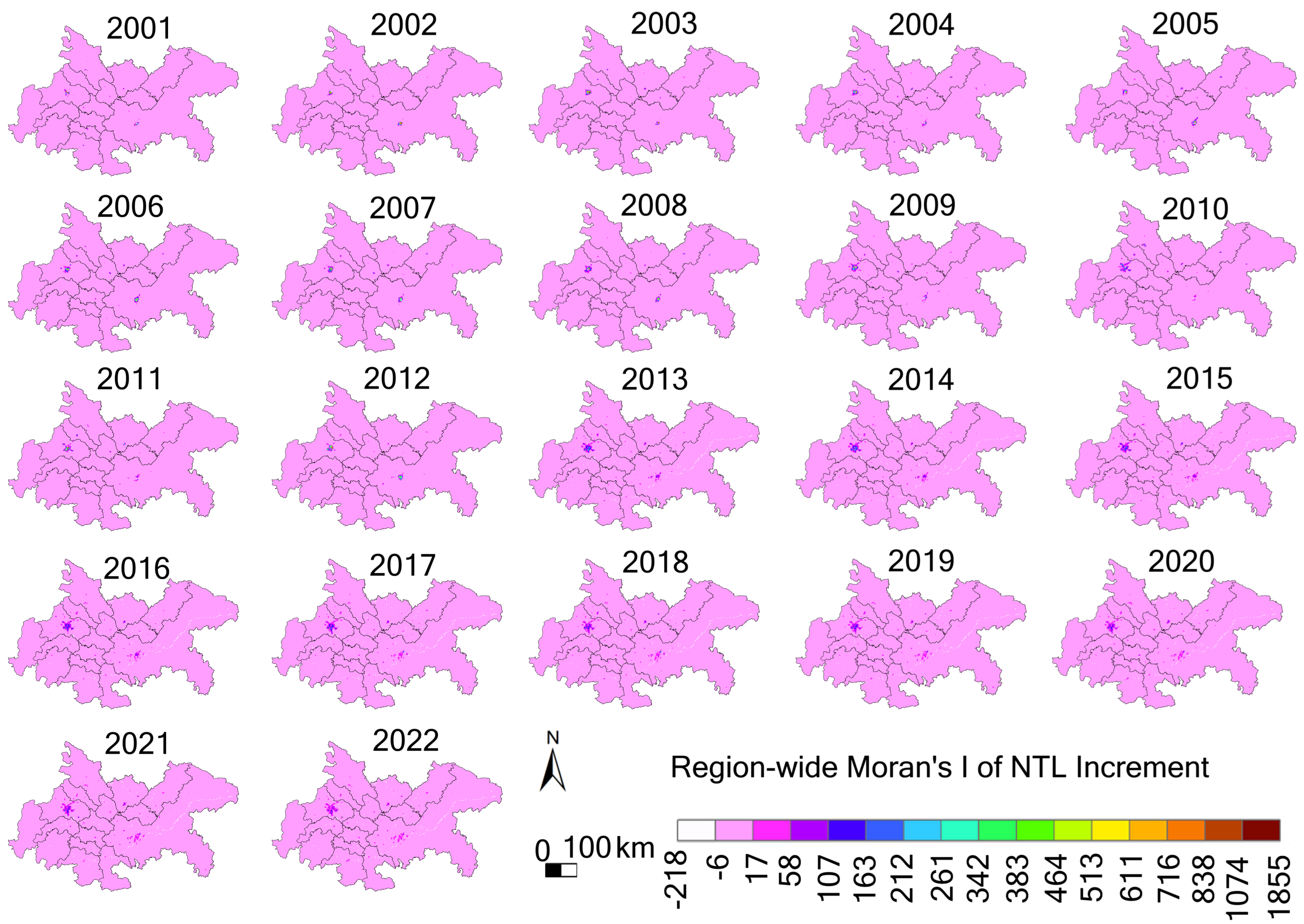


Supplementary Fig. 3 Spatial distribution maps of region-wide Moran's I of NTL Increment in the Chengdu-Chongqing region (2001-2022). Color scale shows each pixel's contribution to global spatial autocorrelation, from low to high values. Values increased markedly after 2013, indicating significantly enhanced spatial clustering intensity.


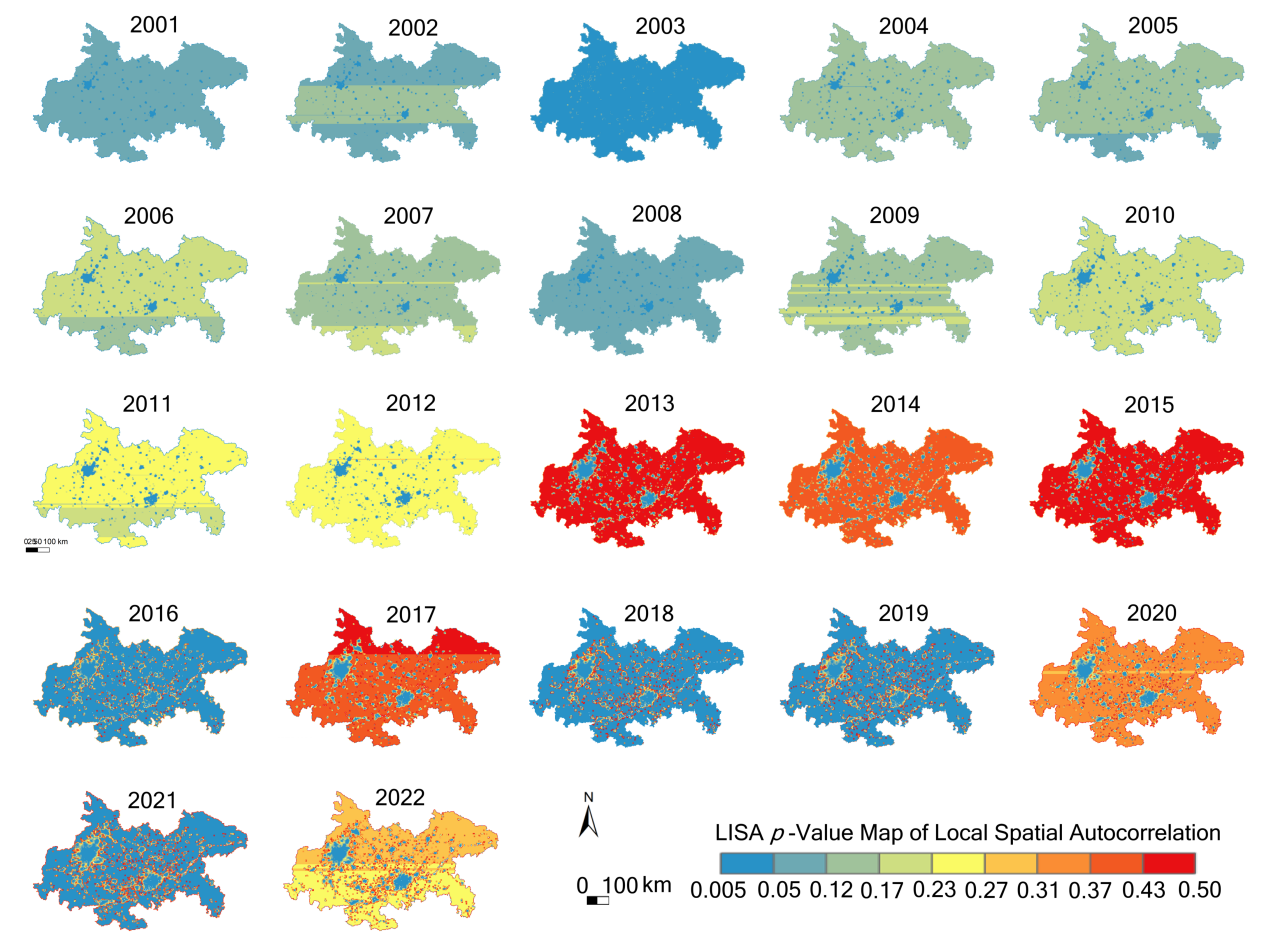


Supplementary Fig. 4 LISA p-value maps of local spatial autocorrelation in the Chengdu-Chongqing region (2001-2022). Color scale indicates statistical significance levels, with low p-value areas representing statistically significant spatial associations (p < 0.05) and high p-value areas representing non-significant spatial patterns.


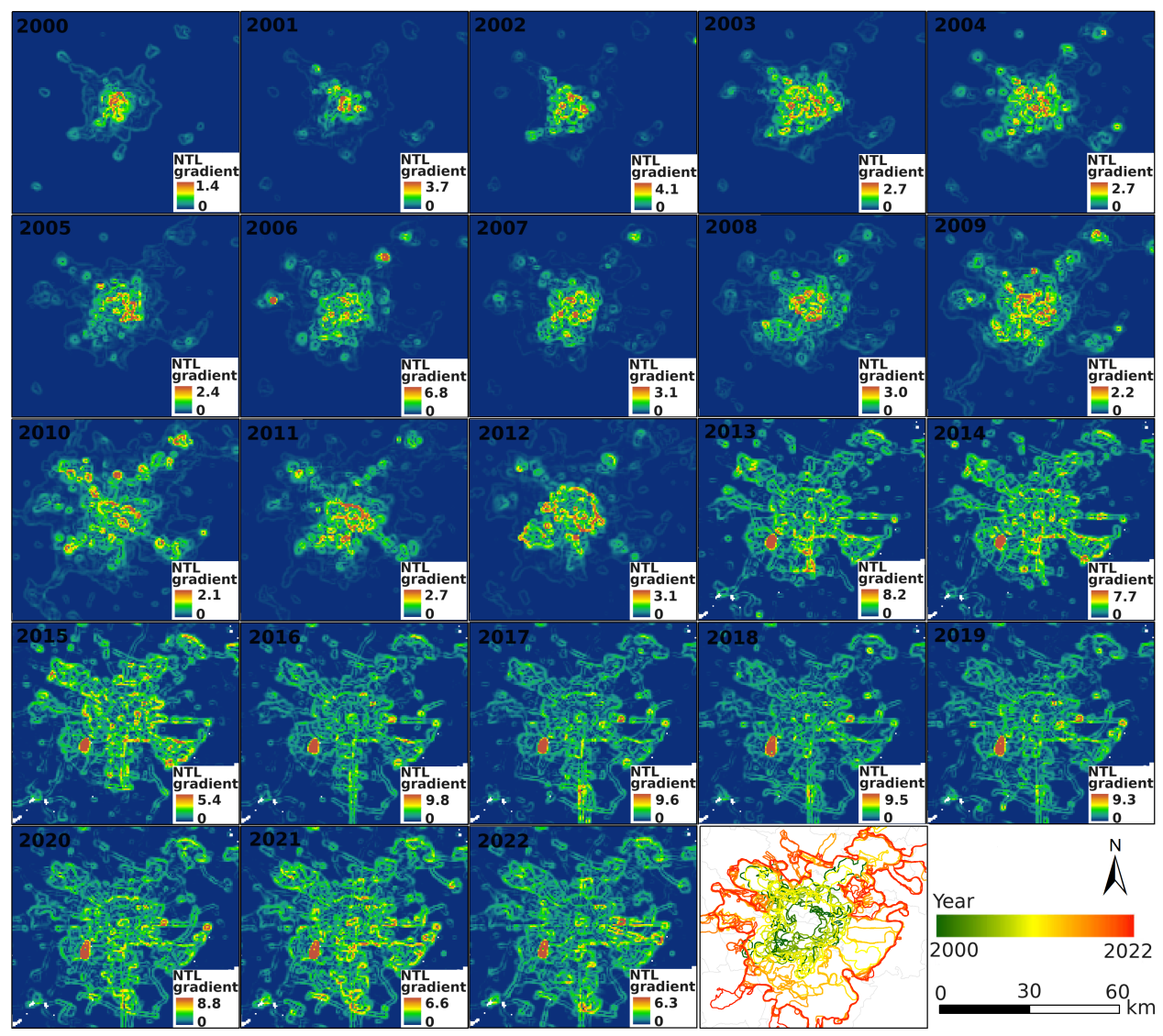


Supplementary Fig. 5 The NTL gradient and derived boundary data of Chengdu from 2000 to 2022. The boundary line is the contour line with a value of 0.2.


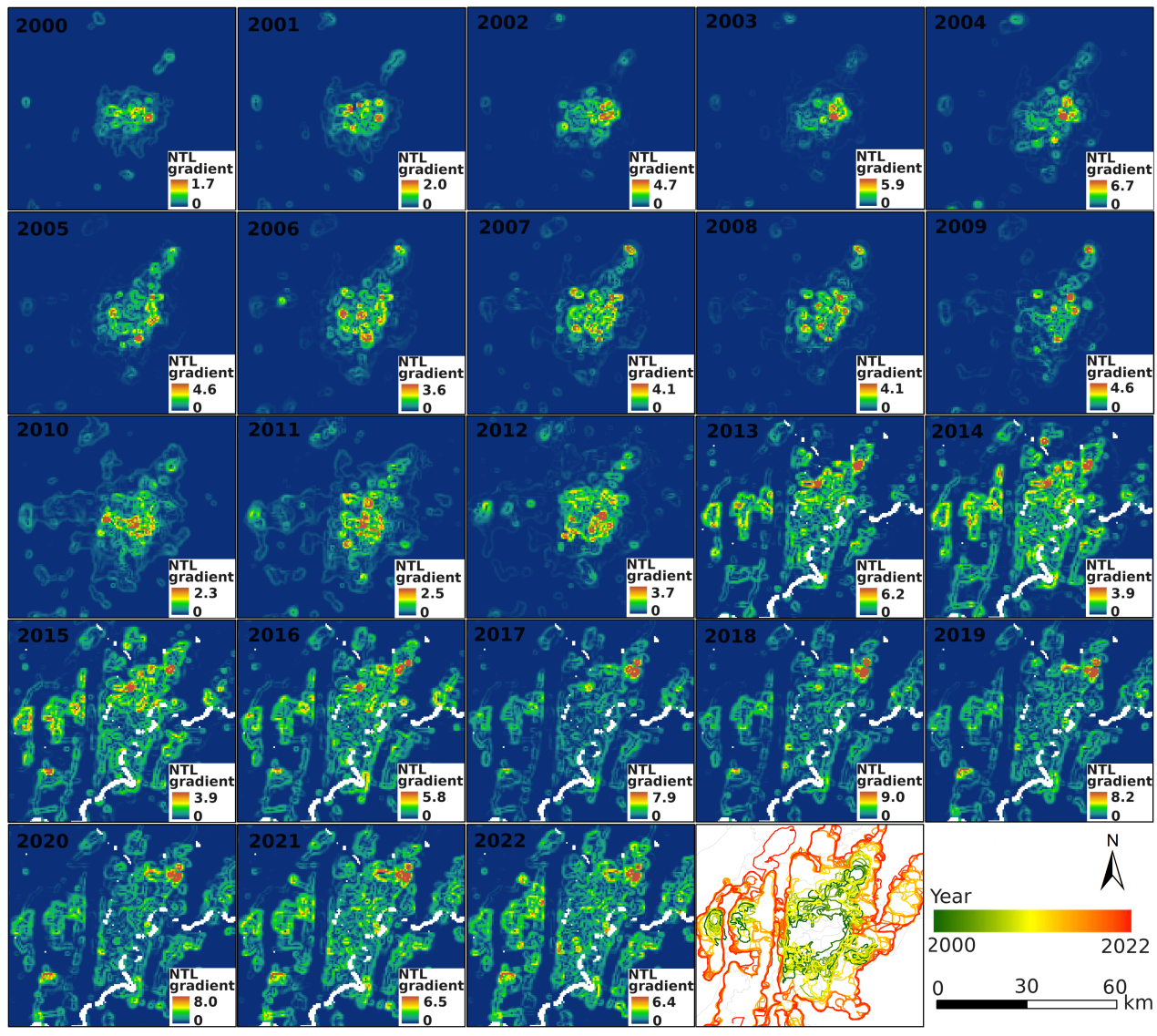


Supplementary Fig. 6 The NTL gradient and derived boundary data of Chongqing from 2000 to 2022. The boundary line is the contour line with a value of 0.1.
